# Ulcerative colitis mucosal transcriptomes reveal mitochondriopathy and personalized mechanisms underlying disease severity and treatment response

**DOI:** 10.1038/s41467-018-07841-3

**Published:** 2019-01-03

**Authors:** Yael Haberman, Rebekah Karns, Phillip J. Dexheimer, Melanie Schirmer, Judith Somekh, Ingrid Jurickova, Tzipi Braun, Elizabeth Novak, Laura Bauman, Margaret H. Collins, Angela Mo, Michael J. Rosen, Erin Bonkowski, Nathan Gotman, Alison Marquis, Mason Nistel, Paul A. Rufo, Susan S. Baker, Cary G. Sauer, James Markowitz, Marian D. Pfefferkorn, Joel R. Rosh, Brendan M. Boyle, David R. Mack, Robert N. Baldassano, Sapana Shah, Neal S. Leleiko, Melvin B. Heyman, Anne M. Grifiths, Ashish S. Patel, Joshua D. Noe, Bruce J. Aronow, Subra Kugathasan, Thomas D. Walters, Greg Gibson, Sonia Davis Thomas, Kevin Mollen, Shai Shen-Orr, Curtis Huttenhower, Ramnik J. Xavier, Jeffrey S. Hyams, Lee A. Denson

**Affiliations:** 1 Cincinnati Children’s Hospital Medical Center, and the University of Cincinnati College of Medicine, 45229, Cincinnati, OH USA; 2 Sheba Medical Center, Tel Hashomer, affiliated with the Tel Aviv University, Tel Aviv, 5265601 Israel; 3 Broad Institute of MIT and Harvard University, Cambridge, 02142 MA USA; 4 Faculty of Medicine, Technion, Haifa, 3109601 Israel; 5 Department of Information Systems, University of Haifa, Haifa, 3498838 Israel; 6 Children’s Hospital of Pittsburgh, Pittsburgh, 15224 PA USA; 7 Department of Pediatrics, University of California at San Diego, La Jolla, 92162 CA USA; 8 Georgia Institute of Technology, Atlanta, 30332 GA USA; 9 Collaborative Studies Coordinating Center, University of North Carolina, Chapel Hill, 27516 NC USA; 10 Harvard—Children’s Hospital Boston, Boston, 02115 MA USA; 11 Women & Children’s Hospital of Buffalo WCHOB, Buffalo, 14222 NY USA; 12 Emory University, Atlanta, 30322 GA USA; 13 Cohen Children’s Medical Center of New York, 11040, New Hyde Park, NY USA; 14 Riley Hospital for Children, Indianapolis, 46202 IN USA; 15 Goryeb Children’s Hospital—Atlantic Health, Morristown, 07960 NJ USA; 16 Nationwide Children’s Hospital, Columbus, 43205 OH USA; 17 Children’s Hospital of East Ontario, Ottawa, Ontario, K1P 1J1 Canada; 18 The Children’s Hospital of Philadelphia, Philadelphia, 19104 PA USA; 19 Children’s Hospital of Pittsburgh of UPMC, Pittsburgh, 15224 PA USA; 20 Hasbro Children’s Hospital, Providence, 02903 RI USA; 21 University of California at San Francisco, San Francisco, 94143 CA USA; 22 Hospital for Sick Children, Toronto, M5G 1X8 Canada; 23 UT Southwestern, Dallas, 75390 TX USA; 24 Medical College of Wisconsin, Milwaukee, 53226 WI USA; 25 RTI International, Research Triangle Park, 27709 NC USA; 26 Harvard School of Public Health, Boston, 02115 MA USA; 27 Massachusetts General Hospital, Harvard Medical School, Boston, 02114 MA USA; 28 Connecticut Children’s Medical Center, Hartford, 06106 CT USA

## Abstract

Molecular mechanisms driving disease course and response to therapy in ulcerative colitis (UC) are not well understood. Here, we use RNAseq to define pre-treatment rectal gene expression, and fecal microbiota profiles, in 206 pediatric UC patients receiving standardised therapy. We validate our key findings in adult and paediatric UC cohorts of 408 participants. We observe a marked suppression of mitochondrial genes and function across cohorts in active UC, and that increasing disease severity is notable for enrichment of adenoma/adenocarcinoma and innate immune genes. A subset of severity genes improves prediction of corticosteroid-induced remission in the discovery cohort; this gene signature is also associated with response to anti-TNFα and anti-α_4_β_7_ integrin in adults. The severity and therapeutic response gene signatures were in turn associated with shifts in microbes previously implicated in mucosal homeostasis. Our data provide insights into UC pathogenesis, and may prioritise future therapies for nonresponders to current approaches.

## Abstract (web summary)

The severity of ulcerative colitis, and response to treatment, is highly variable. Here, the authors examine rectal gene expression signatures and faecal microbiomes of children and adults with the disease and provide new insights in to pathogenesis.

## Introduction

Ulcerative colitis (UC) is a chronic relapsing-remitting inflammatory bowel disease (IBD) diagnosed primarily in young individuals. The disease burden has increased with globalization; newly industrialized countries show the greatest increase in incidence^1^ and the highest prevalence is recorded in Western countries^2^. Disease severity and treatment response are strikingly heterogeneous with some patients quickly and continually responding to initial therapies while others experience ongoing inflammation ultimately requiring surgical resection of the affected bowel^3,4^. Greater understanding of individualized pathways driving clinical and mucosal severity and response to therapy, and the clinical translation of these data, is needed to proactively identify targeted therapeutic approaches.

To improve our understanding of UC pathogenesis and its potential clinical personalized translation, we applied a standardized approach to a large, multicenter inception cohort that collected samples before treatment initiation, and included subjects representing the full spectrum of disease severities. The Predicting Response to Standardized Pediatric Colitis Therapy (PROTECT) study included 428 UC patients from 29 pediatric gastroenterology centers in North America^3^. At diagnosis, disease was clinically and endoscopically graded, rectal biopsy histology was centrally read^5^, and clinical and demographic data were recorded. Patients were assigned a specific standardized initial therapy with mesalamine or corticosteroids, and outcomes were recorded. Rectal biopsies from a representative subcohort of 206 patients underwent high-throughput RNA sequencing (RNAseq) prior to medical therapy, representing the largest UC transcriptomic cohort to date (Supplementary Table 1). We capture robust gene expression and pathways that are linked to UC pathogenesis, severity, response to corticosteroid therapy, and gut microbiota, which provide new insights into molecular mechanisms driving disease course.

## Results

### A unique treatment-naive UC inception cohort

The PROTECT study systematically examined response of 428 newly diagnosed pediatric UC patients to consensus-defined disease severity-based treatment regimens guided by the Pediatric Ulcerative Colitis Activity Index (PUCAI)^3^. mRNAseq defined pretreatment rectal gene expression for a representative discovery group of 206 UC PROTECT patients, a validation group of 50 UC PROTECT patients, and 20 age- and sex-matched non-IBD controls (Table 1). The validation group had similar characteristics to the discovery group, but with a higher frequency of nonwhite participants. More severe endoscopic disease (Grade 3 Mayo endoscopic subscore, Chi squares *p* < 0.001) and more extensive disease or pancolitis (Chi squares *p* < 0.001) were noted in moderate-severe cases. Of the patients with mild disease, 53 (98%) of 54 received initial therapy with mesalamine, and all moderate-severe patients received initial therapy with corticosteroids. Week 4 remission was defined as PUCAI < 10 without additional therapy or colectomy and was achieved by 105 of 206 (51%) patients in the discovery cohort. One hundred and fifty-six patients also had 16S rRNA sequencing to characterize their gut microbial communities.

**Table 1:** Characteristics of controls and PROTECT ulcerative colitis discovery and validation cohorts

	Ctl (*n* = 20) RNAseq	UC (*n* = 428) Full PROTECT Cohort	UC (*n* = 206) RNAseq	UC mild (*n* = 54) RNAseq	UC mod-sev Discovery (*n* = 152) RNAseq	UC mod-sev Validation (*n* = 50) RNAseq
Age (Mean ± SD)	13.9 ± 3.3	12.7 ± 3.3	12.9 ± 3.2	13.1 ± 3.5	12.8 ± 3.1	12.4 ± 3.4
Sex M (%)	9 (45%)	216 (50%)	112 (54%)	32 (59%)	80 (53%)	23 (46%)
BMI *z* score (Mean ± SD)	0.3 ± 1.6	−0.2 ± 1.3	−0.26 ± 1.32	−0.08 ± 1.19	−0.33 ± 1.36	−0.28 ± 1.27
White	17/20 (85%)	351/420 (84%)	204/206 (99%)	52/54 (96%)	152/152 (100%)	28/50 (56%)
PUCAI score (range 0–85)						
10–30 (Mild)	—	102 (24%)	54 (26%)	54 (100%)	—	—
35–60 (Moderate)	—	185 (43%)	84 (41%)	—	83 (55%)	21 (42%)
≥65 (Severe)	—	141 (33%)	68 (33%)	—	69 (45%)	29 (58%)
Mayo endoscopy subscore (range 0–3)						
Grade 1 Mild	—	59 (14%)	27 (13%)	20 (37%)	7 (5%)	2 (4%)
Grade 2 Moderate	—	224 (52%)	108 (52%)	29 (54%)	79 (52%)	22 (44%)
Grade 3 Severe	—	145 (34%)	71 (34%)	5 (9%)	66 (43%)	26 (52%)
Disease location						
Proctosigmoiditis	—	29 (7%)	14 (7%)	11 (20%)	3 (2%)	0 (0%)
Left-sided colitis	—	44 (10%)	25 (12%)	14 (26%)	11 (7%)	1 (2%)
Extensive/Pancolitis/^a^ Unassessable	—	355 (83%)	167 (81%)	29 (54%)	138 (91%)	49 (98%)
Initial treatment						
Mesalamine	—	136 (32%)	53 (26%)	53 (98%)	—	
Oral or IV steroids	—	292 (68%)	153 (74%)	1 (2%)	152 (100%)	50 (100%)
Oral steroids	—	144 (34%)	82 (40%)	1 (2%)	81 (53%)	20 (40%)
IV steroids	—	148 (34%)	71 (34%)	—	71 (47%)	30 (60%)
Week 4 remission (PUCAI < 10)	—	211/422 (50)%	105 (51%)	30 (56%)	75 (49%)	21 (42%)
Week 4 fecal calpro < 250	—	56/282 (20%)	39/150 (26%)	14/42 (33%)	25/108 (23%)	9/28 (32%)

*PUCAI* Pediatric Ulcerative Colitis Activity Index

^a^Unassessable: severe/fulminant disease at presentation and the clinician performed a flexible sigmoidoscopy for safety concerns. Data are mean ± SD, *n* (%), *n*/*N* (%) unless noted otherwise. *n*/*N* values show missing data

### The core UC gene signature

**Fig. 1:**
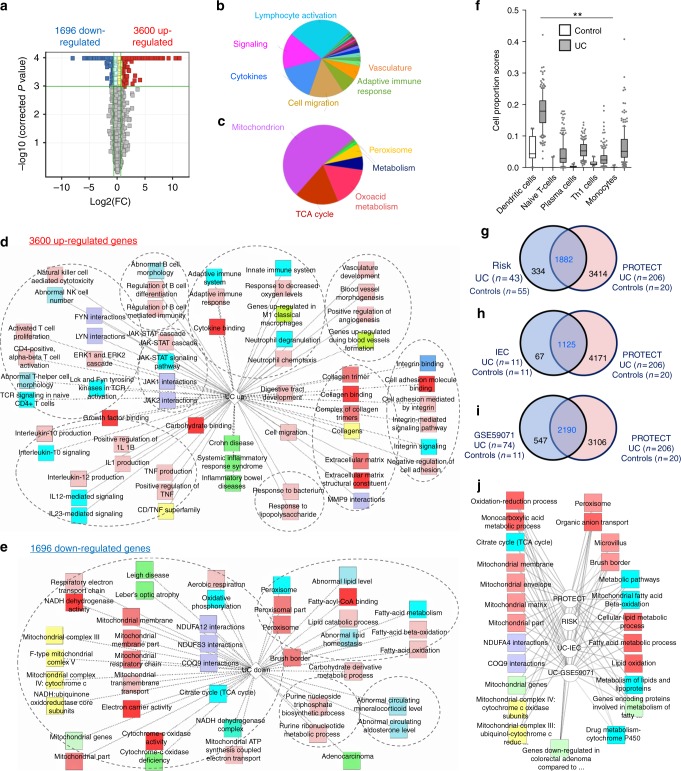
The core genes and pathways of newly diagnosed treatment-naive pediatric Ulcerative colitis emphasize lymphocyte activation and mitochondrial dysfunction. **a** Volcano plot of the 5296 differentially expressed genes between 206 UC and 20 Ctl samples (FC ≥ 1.5 and FDR < 0.001). Functional annotation enrichment analyses of the **b** 3600 upregulated and **c** 1696 downregulated UC core genes using CluGO^8^ charts. Detailed functional annotation enrichment analyses of the **d** 3600 upregulated and **e** 1696 downregulated UC core genes using ToppGene^6^, ToppCluster^7^, and Cytoscape^52^ are shown. GO Biological Process, Cellular Component, and Molecular Function (pink), pathways (light blue), mouse phenotype (blue), gene family (yellow), coexpression (light green), disease (dark green), interactions (purple). The full list of gene set enrichment results and *P* values are in Supplementary Dataset 1. **f** Computational deconvolution of cell subset proportions in 206 UC and 20 controls. Differences (Wilcoxon test with FDR < 0.01 (**)) are shown for cell types with at least 80% non-zero values. Overlap of differentially expressed genes between UC and Ctl in **g** RISK, **h** isolated colon epithelial cells (IEC^13^), and **i** a microarray study of adult cases (GSE59071 ^12^). **j** Detailed functional annotation enrichment analyses of the shared downregulated functions of the above studies. Box and whisker plot with central line indicating median, box ends representing upper and lower quartile, and whisker represent 10–90 percentile. *UC* ulcerative colitis

We defined a core rectal UC gene expression signature composed of 5296 genes (Fig. 1a) differentially expressed (FDR < 0.001 and fold change (FC) ≥ 1.5) in comparison to controls (Ctl, Fig. 1 and Supplementary Dataset 1). Functional annotation enrichment analyses using ToppGene^6^, ToppCluster^7^, and CluGO^8^ mapped groups of related genes to biological processes^9^. Overview CluGo pie charts (Fig. 1b, c) showed highest enrichment for increased lymphocyte activation and associated cytokine signaling, and a robust decrease in mitochondrion, aerobic tricarboxylic acid (TCA) cycle, and metabolic functions. *P* values for the top specific biological processes were obtained as an output from ToppGene (Supplementary Dataset 1) and more detailed ToppCluster pathways analysis output is shown in Fig. 1d for the 3600 upregulated and Fig. 1e for the 1686 downregulated genes. Upregulated gene signatures were enriched for integrin signaling (*P* < 1.08E-12), JAK-STAT cascade, and TNF production (*P* < 9.9E-93), pathways that are already associated with therapeutic advances in UC^10,11^. The downregulated UC signature showed a robust decrease of mitochondrial-encoded and nuclear-encoded mitochondrial genes (*P* < 2.76E-35). Applying a computational gene expression deconvolution approach to estimate the relative composition of immune cell subsets, epithelia, and other stromal cell types in each sample (Methods) showed a significant increase in the estimated proportion of several immune cells including T and B cells, dendritic cells (DC), and monocytes (Fig. 1f). Using RISK cohort rectal biopsies mRNAseq data for treatment-naïve pediatric UC patients (Fig. 1g, Supplementary Dataset 1) and colonic biopsies microarray data of adults with active UC (Fig. 1i, Supplementary Dataset 1, GSE59071^12^), we demonstrate that 87% of the differentially expressed genes in RISK UC, and 80% of the adult UC genes, were within our core PROTECT signature. Comparing the differentially expressed genes from isolated intestinal epithelial cells (IEC) from another pediatric UC inception cohort^13^ showed an overlap of 94% of the genes identified in that study (Fig. 1h) with the PROTECT genes, validating the majority of the core PROTECT UC signature in whole biopsies and in isolated epithelia. Functional annotation enrichment analyses of the shared genes further confirmed many of the common enriched pathways (Supplementary Dataset 1). Comparing the shared downregulated genes and pathways between PROTECT, RISK, adult UC cohort GSE59071^12^, and the IEC UC cohort^13^ using ToppGene/ToppCluster confirmed the reduction of mitochondrial metabolic-associated genes and pathways, genes associated with lipid metabolism, and genes associated with formation of adenoma and adenocarcinoma (Fig. 1j).

### Robust colonic mitochondriopathy in UC

**Fig. 2:**
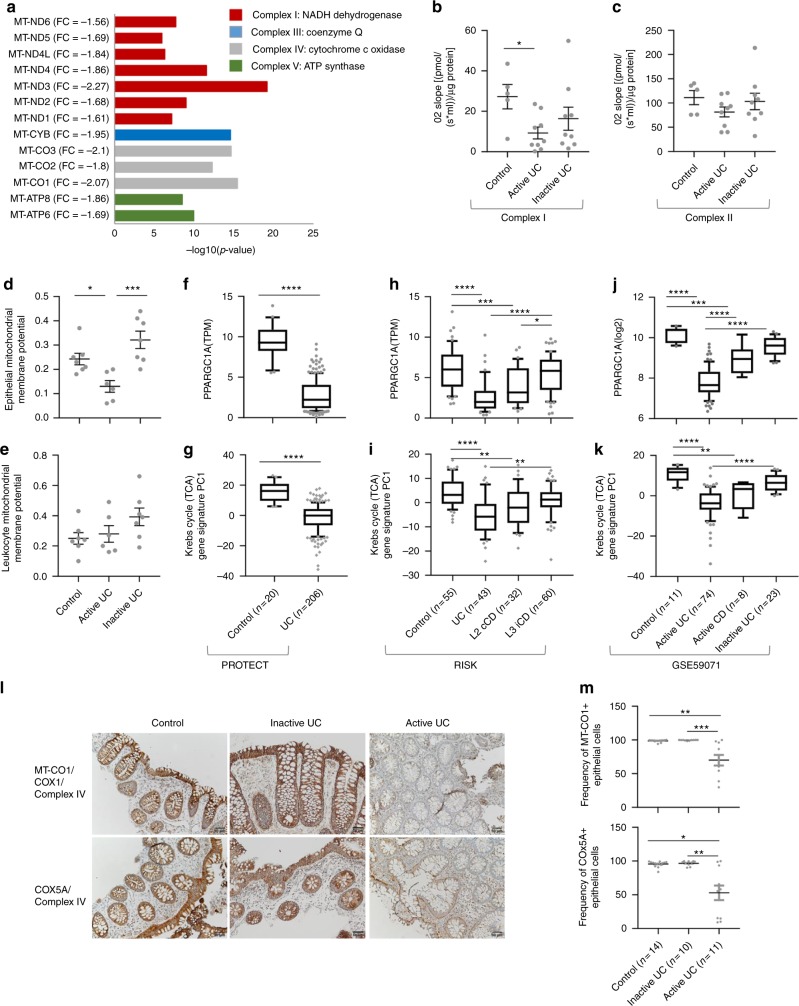
Colonic mitochondriopathy with a robust gene signature for reduced rectal mitochondrial energy functions in UC. **a** Thirteen mitochondrial-encoded genes are downregulated in UC vs. control with their fold change, FDR corrected *P* value, and associated mitochondrial complex as indicated. High-Resolution Respirometry was performed on fresh colon biopsies (5 control, 9 with active UC, and 9 with inactive UC) using the Oroboros O2k modular system to evaluate the activity of Complex I (**b**) and Complex II (**c**) of the electron transport chain. JC1 staining and FACS analysis were used to define the mitochondrial membrane potential of **d** EpCAM + epithelial cells and **e** CD45 + leukocytes isolated from colon biopsies (7 controls, 6 active UC, and 7 with inactive UC, 85–99% viability). Colon PPARGC1A (PGC-1α) expression for **f** PROTECT cohort, **h** RISK cohort in (transcripts per million (TPM) values), and for **j** adult UC cohort (GSE59071^12^) in normalized values was plotted after stratifying the samples as indicated. **g**, **i**, **k** Krebs cycle TCA gene signature PCA PC1 for the above cohorts is plotted, samples are stratified as indicated. **l** Representative rectal MT-CO1 and COX5A immunohistochemistry (complex IV) for Ctl (*n* = 14), inactive (*n* = 10), and active UC (*n* = 11) with moderate Mayo endoscopic subscore and moderate PUCAI. Scale bar represents 50 μm. **m** Frequency of MT-CO1-positive and COX5A-positive epithelial cells out of the total epithelial cells for controls, inactive UC, and active UC. Lines in the scatter dot plots represent mean and SEM. Kruskal−Wallis with Dunn’s Multiple Comparison or ANOVA with false discovery rate (FDR) was used. *All two-sided *P* < 0.05, **P < 0.01, ***P < 0.001, ****P < 0.0001. UC ulcerative colitis, L2 cCD colon-only Crohn’s disease L3 iCD ileo-colonic Crohn’s disease

Notably, the mitochondrial genome encodes 13 genes regulating ATP production and all 13 were significantly reduced in UC (Fig. 2a). Real-time analysis of cellular respiration^14^ was subsequently evaluated in colonic biopsies from UC and control patients. Mitochondrial electron transport chain Complex I activity, the rate-limiting step in oxidative phosphorylation^15,16^ was reduced in active UC rectal biopsies compared to those from control patients (Fig. 2b). There was also a nonsignificant trend toward a decrease in Complex II activity (Fig. 2c). The mitochondrial membrane potential (MMP) that provides an integrated measure of the cellular capacity for ATP production was measured using JC-1 staining and FACS analysis of freshly isolated EpCAM+ colon epithelial cells (Fig. 2d) and CD45+ leukocytes (Fig. 2e). This showed a specific reduction in epithelial cells of active UC, with recovery in inactive UC (Supplementary Fig. 1). In addition, *PPARGC1A* (PGC-1α), the master regulator of mitochondrial biogenesis, was profoundly reduced in UC patients in comparison to controls in PROTECT, RISK, and adult UC (Fig. 2f, h, j), and the IEC UC cohort (Supplementary Dataset 1)^13^. Principal coordinates analysis (PCA) principal components 1 (PC1) to summarize the Krebs cycle (TCA) genes variations between patients showed reduction of genes regulating mitochondrial energy production in the UC groups (Fig. 2g, i, k). The RISK dataset revealed a spectrum of mitochondrial gene expression downregulation in inflamed whole rectal biopsies, ranging from no significant suppression in mucosal biopsies obtained from inflamed rectum of ileo-colonic CD (L3 iCD) patients, to moderate suppression in samples from inflamed rectal biopsies of colon-only CD (L2 cCD) patients, and profound suppression in samples from pediatric UC samples with inflamed rectum (Fig. 2h, i). The spectrum between UC and CD was validated in the adult IBD cohort (GSE59071^12^, Fig. 2j, k), and we noted a recovery of this pathway in inactive adult UC. However, the larger PROTECT mRNAseq cohort permitted identification of an additional 3106 differentially expressed genes, which primarily demonstrated more robustly the suppression of mitochondrial pathways (Supplementary Dataset 1). Immunohistochemistry confirmed reduced epithelial abundance of both mitochondrial-encoded MT-CO1 and nuclear-encoded COX5A genes which comprise complex IV in active UC (Fig. 2l, m).

### Disease severity gene signatures

**Fig. 3:**
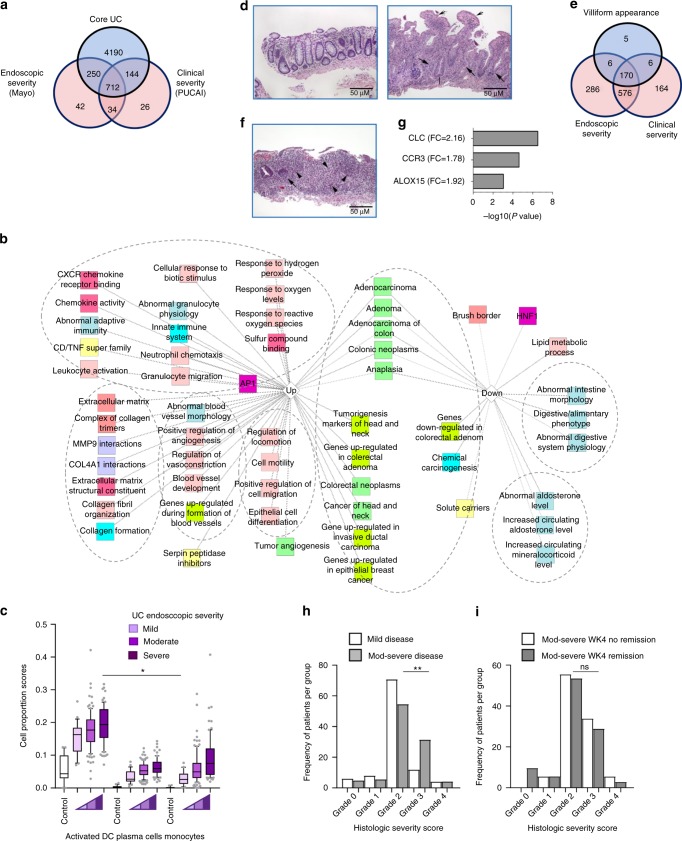
Disease severity is linked to adenoma/adenocarcinoma and innate immune pathways. **a** Venn diagram shows the 712 UC severity genes overlapping the 5296 core UC signature with the 916 clinical severity and 1038 endoscopic severity genes differentially expressed between severe and mild cases (FC ≥ 1.5, FDR < 0.001). **b** Functional annotation enrichment analyses of the 712 UC severity genes. The full list of gene set enrichment results and *P* values are in Supplementary Dataset 2. Node colors are as in Fig. 1. **c** Computational deconvolution of cell subset proportions in controls and UC patients stratified by endoscopic severity Mayo subscore. Differences (ANOVA with FDR < 0.05 (*)) between Mayo 3 (severe, *n* = 71) and 1 (mild, *n* = 27) are shown for those cell types in Fig. 1. **d** Hematoxylin and eosin (H&E, 100×) staining of control (left) and UC (right) case with acute cryptitis (arrows), crypts that do not rest on the muscularis mucosa (bar), and marked surface villiform change (concave arrows). **e** Venn diagram shows the 187 villiform changes genes overlapping with severity genes. **f** H&E staining of UC case with acute cryptitis (arrow) and numerous eosinophils in the lamina propria (arrowheads). **g** Three genes that are associated with presence of >32 eosinophils/HPF in UC. **h** Frequency (percent of patient of the total per group) of mild (*n* = 54) and moderate-severe (*n* = 152) patients across histology severity scores (defined in Supplementary Table 2). **Chi squares *p* < 0.01. **i** Distribution of moderate-severe patients who did or did not achieve week 4 (WK4) remission across histology severity scores. UC ulcerative colitis. Box and whisker plot with central line indicating median, box ends representing upper and lower quartile, and whisker represent 10–90 percentile. Scale bar represents 50 μm

More severe disease is linked in our data and others to higher rates of therapy escalation and colectomy^3,17^, whereas mild disease is associated with remission by 12 weeks. Unsupervised hierarchical clustering analysis using the core 5296 genes grouped 204 of 206 UC cases in the dendrogram cluster A while all 20 non-IBD controls were in cluster B (Supplementary Fig. 2). Most mild cases grouped in A(i), while severe cases tended to be enriched in cluster A(ii) (Supplementary Fig. 2B, *P* < 0.001). The core UC 5296 gene principle component 1 (PC1) values separated Ctl from UC across both clinical and endoscopic severity (Supplementary Fig. 2C, D), while PC2 contributed to separation within UC severity. In all, 106 genes were significantly differentially expressed between severe vs. moderate and between moderate vs. mild UC clinical disease defined by PUCAI, showing stepwise alteration across cases (Supplementary Fig. 2E). We identified 916 genes that differed between UC with severe vs. mild clinical disease and 1038 genes that differed between severe vs. mild endoscopic subscore (FDR < 0.001 and FC ≥ 1.5, Supplementary Dataset 2). The Venn diagram (Fig. 3a) shows the overlap of the resulting 712 genes (292 down- and 420 upregulated genes) and the core UC signature, referred to hereafter as the UC severity signature. Functional annotation enrichment analyses of the UC severity signature (Supplementary Dataset 2, Fig. 3b) emphasized genes that are down- (*P* < 4.54E-46) and upregulated (*P* < 7.62E-51) in colorectal adenoma. Immunohistochemistry (Supplementary Fig. 3) confirmed increased epithelial abundance of *REG1A* gene, known to be upregulated in both UC and in colitis-associated colorectal cancer (CAC)^18^ in active UC. In addition, upregulated severity genes were also enriched for innate immunity (*P* < 7.07E-19), neutrophil degranulation (*P* < 1.51E-16), and CXCR1 interactions (*P* < 9.08E-8). Relative composition of immune cell subsets using a computational gene expression deconvolution approach showed an increase in activated DC, plasma cells, and monocytes in patients with severe vs. mild disease (Fig. 3c). An alternative analytic approach using the Immunological Genome Project data series as a reference through ToppGene^6^ also identified an increased proportion of myeloid cells with increased severity (Supplementary Fig. 4).

### Rectal genes correlated with histologic features

Rectal biopsy histology was evaluated centrally. Surface villiform architectural abnormality (Fig. 3d) was linked to escalation therapy or colectomy in our recent report^3,5^. We identified 187 genes (69 up- and 118 downregulated, Supplementary Dataset 3) that differed (FDR < 0.001 and FC ≥ 1.5) between UC patients with and without surface villiform changes. Most of these genes overlapped with the 712 UC severity genes (Fig. 3e) suggesting a molecular link between this histologic feature and UC severity (Supplementary Dataset 3). In contrast, higher eosinophil infiltrate (>32 rectal eosinophils/hpf, Fig. 3f) was associated with a favorable week 12 outcome^3,5^. Three genes differed significantly (FDR < 0.001 and FC ≥ 1.5) between UC patients with and without higher infiltrating eosinophils (Fig. 3f, g). This included Arachidonate 15-Lipoxygenase (*ALOX15*) involved in production of lipid mediators which resolve inflammation. A Histologic Severity Score (HSS) for chronic and active acute neutrophil inflammation was defined^5^ (Supplementary Table 2). While we noted a higher frequency of patients with moderate-severe disease showing marked acute inflammation with crypt abscesses (grade 3) histology than the frequency noted within patients with mild disease (Fig. 3h), no such difference was noted within moderate-severe patients that did or did not achieve week 4 (WK4) remission (Fig. 3i).

### Corticosteroid response gene signature and microbial shifts

**Fig. 4:**
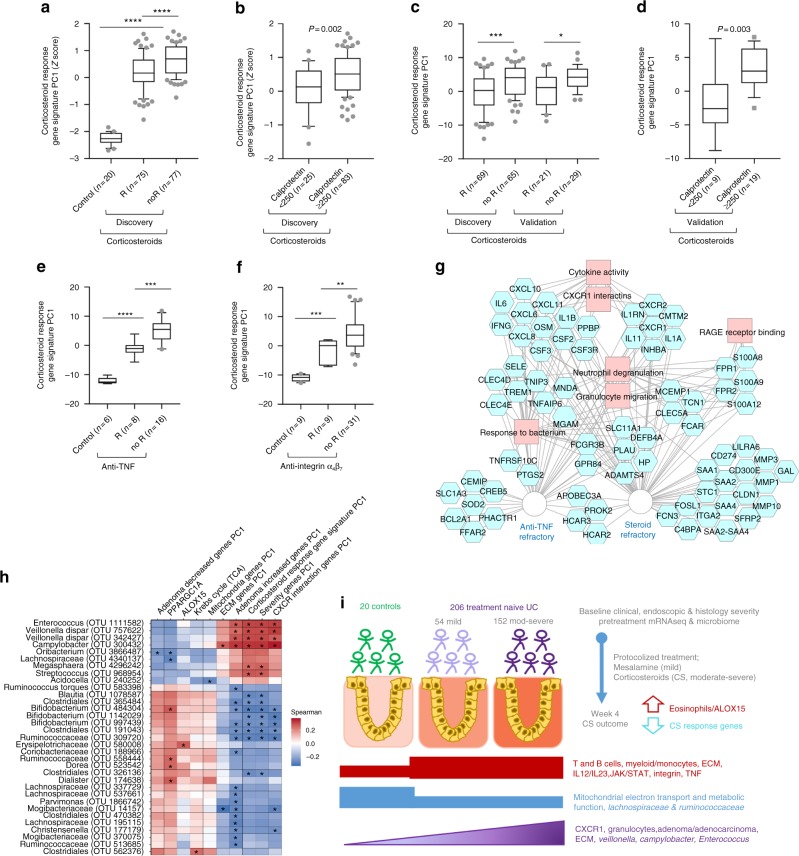
A rectal gene signature is associated with response to UC induction therapy and microbial shifts. Samples loading PC1 (Z score) values of the corticosteroid response gene signature are shown for controls and the discovery cohort of 152 moderate-severe UC patients stratified by **a** WK4 clinical remission (R) and **b** mucosal healing (fecal calprotectin < 250 mcg/gm). Samples loading PC1 values derived from an independent 3′UTR Lexogen mRNASeq platform are shown for the discovery cohort and an independent validation cohort stratified by **c** WK4 clinical remission (R), or by **d** mucosal healing as in (**b**) for the validation cohort. Samples loading PC1 values including controls and **e** the GSE16879^20^ data set of UC treated with anti-TNF and **f** GSE73661^23^ of UC treated with anti-integrin α_4_β_7_. R: mucosal healing defined by colonoscopy. **g** Functional annotation enrichment analyses of the corticosteroid response gene signature and the top 50 genes that were differentially expressed in pretreatment colon biopsies of anti-TNF refractory vs. responsive UC patients^21^. The full list of enriched functions and *P* values are in Supplementary Dataset 4. Genes are in blue and biologic functions in pink; connections to each signature are as shown. **h** Heat map summarizing Spearman similarity measures between microbial abundances and gene expression using hierarchical all-against-all association. *False discovery rate < 0.2. Blue and red indicate negative and positive associations, respectively. **i** Graphical summary of the cohort and main findings. Box and whisker plot with central line indicating median, box ends representing upper and lower quartile, and whisker represent 10–90 percentile. Mann−Whitney and ANOVA FDR as applicable, *P < 0.05, **P < 0.01, ***P < 0.001, ****<0.0001

In the full cohort, the strongest predictor of corticosteroid-free remission by week 12 was clinical remission at week 4 (WK4), irrespective of initial corticosteroid status^3^. When considering WK4 remission, clinical factors associated with this outcome included disease severity and rectal biopsy eosinophil count^3^. Based on these results, we focused our analysis on the WK4 outcome of moderate-severe patients that received corticosteroids. A corticosteroid response gene signature composed of 115 differentially expressed genes (FDR < 0.05 and FC ≥ 1.5) in baseline rectal biopsies between moderate-severe UC patients who did or did not achieve WK4 remission was defined (Fig. 4, Supplementary Dataset 4, and Supplementary Fig. 5). PCA PC1 values summarized variation in the corticosteroid response gene signature which was differentially expressed based on Week 4 clinical remission (R vs. NoR, Fig. 4a), and week 4 mucosal healing defined as fecal calprotectin <250 mcg/gm (Fig. 4b) in the Illumina discovery cohort. Healthy controls showing lower scores, implying that patients destined to respond to CS have a more healthy profile with respect to this gene signature at baseline. The corticosteroid response gene signature PC1 was replicated using the Lexogen platform^19^ in the subset of 134 UC patients with Illumina data, as well an independent subcohort of 50 UC patients that were not included in the original analysis (Fig. 4c, d). As there are no other mucosal transcriptomic studies that examined response to standardized initial corticosteroid induction therapy, we tested previous transcriptomic studies that examined anti-TNF (GSE16879^20^, used by refs. ^21,22^) or anti-integrin α_4_β_7_ (GSE73661^23^) response. We noted a similar difference with anti-TNF or anti-integrin α_4_β_7_ response in adult UC, in this case defined by mucosal healing at colonoscopy (Fig. 4e, f).

Interestingly, Oncostatim M (OSM^21^) and TREM1^22^ previously associated with anti-TNF response were within our corticosteroid response gene signature (Fig. 4g), and this signature PC1 showed a high correlation with OSM and TREM1 (0.79 and 0.89, *P* < 0.0001). We also noted a substantial overlap between the genes from the PROTECT corticosteroid response gene signature and previously described anti-TNF response genes^21^ (Fig. 4g). Functional annotation enrichment analyses of the corticosteroid response gene signature were performed and the full output from ToppGene (Supplementary Dataset 4) with more detailed ToppCluster output is shown in Fig. 4g. Those analyses indicated that this signature is highly associated with chemokines including CXCR (*P* < 7.12E-12), innate myeloid immune signatures (*P* < 1.62E-15), and response to bacteria (*P* < 2.16E-13). Aberrant immune responses to shifts in commensal microbes likely play a role in UC pathogenesis and treatment responses. In all, 152 of the 206 UC patients in our cohort also had fecal 16S rRNA microbial profiles^24^. By applying hierarchical all-against-all association testing (HAllA: http://huttenhower.sph.harvard.edu/halla), we identified genes and pathways associated with specific microbial operational taxonomic units (OTUs), including associations between disease severity associated taxa such as *Campylobacter*, *Veillonella*, and *Enterococcus* with genes and pathways linked to a more severe disease form, and refractory disease in connection with initial corticosteroid induction therapy. In contrast, we identified decreased taxa from the Clostridiales order that are considered beneficial, which show a negative correlation with gene signatures associated with disease severity and unfavorable treatment responses (Fig. 4h and Supplementary Dataset 5).

### A gene signature improves prediction of week 4 remission

We next asked whether gene expression data would improve a multivariable regression WK4 prediction model based on clinical factors alone (Table 2 and Supplementary Table 3). A model that included (Table 2, model 1) sex, disease severity (total Mayo clinical and endoscopic severity score), and histologic characterization of rectal eosinophils agreed with the model for the full cohort, adding sex with borderline significance. The corticosteroid response gene signature PC1 was negatively associated with week 4 outcome (model 2, OR 0.36, 95% CI 0.18–0.71; *P* = 0.003). When this gene signature was included, the AUC improved to 0.774 (Likelihood ratio *P* value < 0.002), indicating superiority to the model which included clinical factors alone. In model 3, we replaced the eosinophil count with the eosinophil-associated gene *ALOX15* without harming the model accuracy with some improvement of the discriminant power (AUC of 0.777, 0.692−0.848), sensitivity of 62.7%, (95% CI 52.8–72.5%), specificity of 76.6% (95% CI 0.68.8−84.4%), positive predictive value of 72.3%, and negative predictive value of 67.8% (AUC cutoff at ≥0.5). Bootstrapping and multiple imputation were used for internal validation and were generally supportive of the final selected moderate/severe model (Supplementary Fig. 6). The HSS showed moderate correlation with the corticosteroid response gene signature PC1 (Spearman *r* = 0.31, *P* < 0.001), but not with WK4 outcome. Moreover, the gene signature was still significant in the model even after adjusting for the HSS (Supplementary Table 3). Similarly, while the monocyte deconvolution score showed high correlation with the corticosteroid response gene signature PC1 (Pearson *r* = 0.72, *P* < 0.001) and was different between WK4 responders and nonresponders (Supplementary Fig. 5), it was not significant when added to the model in place of the gene signature, while the gene signature remained significant in the model after adjusting for the monocyte score (Supplementary Table 3).

**Table 2:** Multivariable models of baseline characteristics and gene expression associated with week 4 remission in 147 patients with moderate-severe disease that received corticosteroids

Model #	Model variables	OR (95% CI)	Variable *P*	Model AIC	Model AUC	Model ChiSq	Model *P*
1	Total Mayo score (range 0–12) Rectal eosinophil level (count > 32 /hpf) Sex (M vs. F)	0.68 (0.54–0.85) 2.27 (1.11–4.63) 0.47 (0.23–0.96)	0.0007 0.0245 0.039	186.03	73.7 (65.4–82.0)	25.75	<0.0001
2	Total Mayo score (range 0–12) Rectal eosinophil level (count > 32 /hpf) Sex (M vs. F) Corticosteroid response gene signature (PC1 *z*-score values)	0.77 (0.61–0.98) 1.81 (0.85–3.84) 0.47 (0.22–0.99) 0.36 (0.18–0.71)	0.032 0.122 0.048 0.003	178.51	77.4 (69.7–85.1)	35.27	<0.0001
3	Total Mayo score (range 0–12) ALOX15 gene exp. (TPM) Sex (M vs. F) Corticosteroid response gene signature (PC1 *z*-score values)	0.79 (0.63–1.00) 2.59 (1.21–5.52) 0.45 (0.21–0.96) 0.40 (0.2–0.79)	0.055 0.014 0.038 0.009	172.98	77.7 (70.0–85.4)	40.80	<0.0001

LR = 9.519 and LR *P* value = 0.002 when comparing model 2 to model 1

*OR* odds ratio, *AIC* Akaike’s information criterion, *AUC* area under the ROC curve, *LR* likelihood ratio, *ROC* receiver operator characteristic

## Discussion

PROTECT is the largest prospective inception cohort study to examine factors associated with early responses to standardized first-line therapy in pediatric UC. We provide evidence for core host gene expression profiles driving lymphocyte activation and cytokine signaling which are targeted by current therapies. However, our data also suggest a robust reduction in epithelial mitochondrial genes and associated energy production pathways in UC, not directly addressed by current approaches. This reduction of mitochondrial genes was validated in treatment-naïve pediatric UC, adults with active UC with longstanding disease, and more specifically in viable isolated epithelia of treatment-naïve pediatric UC. We capture genes and pathways that are linked to UC severity and prioritize those regulating epithelial transformation and innate CXCR-mediated leukocyte recruitment. We identified a gene signature linked to corticosteroid response, which was validated in an independent subset of UC patients, and showed substantial overlap with genes previously associated with anti-TNF response. A multivariable analysis combining the corticosteroid response gene signature PC1 and *ALOX15* gene expression with clinical variables better predicted corticosteroid responsiveness than clinical factors alone. These findings are summarized in Fig. 4i.

Decreased mitochondrial activity^25,26^ was previously described in UC, but understanding of the molecular mechanism was lacking^27–30^. Dysfunctional mitochondria exacerbate barrier dysfunction and inflammation, while pro-^29^ and anti-^30^ inflammatory stimuli affect mitochondrial metabolic functions. *PPARGC1A* (PGC1α), the master regulator of mitochondrial biogenesis, ameliorated experimental colitis, whereby intestinal epithelial depletion of PGC1α suppressed mitochondrial function and the intestinal barrier^31^. Mitochondrial loss also preceded the development of colonic dysplasia in UC^32^, and high mitochondrial activity reflecting electron transport in the ileum was also associated with protection against CD progression in RISK^33^. Our findings of a substantial suppression of all 13 electron transport mitochondrial-encoded genes (Complex I, III, IV, and V), *PPARGC1A* (PGC1α), and epithelial MMP further support the robustness of the colonic mitochondriopathy in UC. Moreover, we demonstrate specificity of mitochondrial gene expression downregulation in colon-only forms of IBD rather than in CD patients with both ileal and colonic inflammation. Interestingly, previous studies in infectious colitis^34^or diverticulitis^35^ demonstrated an induction of immune and wound-healing genes, with considerable overlap with the immune and wound-healing genes identified in pediatric UC for the current report. However, these studies did not demonstrate a similar reduction in mitochondrial genes, suggesting specificity of this response in UC. Functionally, we observe a decrease in the activity of Complex I of the electron transport chain in the inflamed rectums of patients with UC, and a reduction of mitochondrial depolarization more specifically in epithelia. Although a defect in respiration has been observed in the colons of UC patients previously, to our knowledge mitochondrial function from intestinal biopsies has never before been evaluated via high-resolution respirometry. With real-time analysis of intact human tissue, this technique offers precise evaluation of mitochondrial membrane integrity and oxidative capacity. In conjunction with our expression data, these results suggest a downregulation and dysfunction of mitochondrial respiration, characterized by a defect at Complex I, the rate-limiting step in oxidative phosphorylation. Supplementing the mitochondrial electron transport axis via medical, environmental, or nutritional approaches can be potential targets for future therapies.

Inflammation has a substantial cumulative role in colitis-associated colorectal cancer (CA-CRC) development and is closely linked to the extent^36^, duration^37^ and severity^38^. Studies in the noncancerous IBD mucosa indicated that colorectal cancer development in IBD begins many years before the development of neoplasia as part of the occult evolution within the inflamed bowel^39^. Here, we detect a profound dysregulation of gene sets associated with disease severity previously implicated in adenocarcinoma. Our results therefore show that not only at the genomic and epigenetic level^39,40^, but also at the transcriptomic level, already at diagnosis, genes and pathways that are associated with UC severity show associations with epithelial transformation.

Microbial organisms and products affect host immune education, development and response, and aberrant immune responses to commensal microbes likely contribute to gut inflammation which is the hallmark of UC^41^. We identified positive associations between genes and pathways associated with UC severity and response to treatment and disease-linked microbial taxa. Negative associations involved more beneficial commensal taxa with pathways and genes that were linked to resolution of inflammation or upregulated in non-IBD controls. Those included oral pathobionts *Veillonela dispar*, and *Campylobacter*, and depletion of several commensal organisms such as Lachnospiraceae, *Bifidobacterium*, and Ruminococcaceae suggesting a substantial depletion of SCFA-producing bacteria that may affect epithelial barrier function^42^. Those associations will need to be validated in an independent cohort.

In our study^3^ and in previous studies in children and adults^4,43,44^, higher baseline disease severity identified patients less likely to achieve remission with corticosteroids. We supplement and improve those models by adding baseline gene expression data. We identified a gene signature linked to corticosteroid response and validated it in an independent subset of UC patients. The corticosteroid response gene signature is enriched for cytokines and chemokines (*CXCR1/2* and *CXCL/6/8*/10/11/17), which promote activation of the innate immune system and recruitment of neutrophils, and to response to external stimuli and bacteria. Notably, the corticosteroid response gene signature showed a substantial overlap with genes previously associated with anti-TNF response, and exhibited a similar difference between responders and nonresponders to anti-TNF or anti-integrin α_4_β_7_ therapies. These similarities support an emerging concept in the field that the mucosal inflammatory state as measured by gene expression may better define the likelihood of response to current treatment approaches than conventional clinical measures of severity. By comparison, higher *ALOX15* expression was linked to a higher likelihood for remission. Increasing evidence suggests a role for *ALOX15* expressed in tissue eosinophils and macrophages in the resolution of inflammation^45^, by interfering with neutrophil recruitment in models of arthritis^46^, postoperative ileus^47^, and peritonitis^48^.

Our study has several strengths, but also some limitations. We utilized highly sensitive sequencing and novel analytic approaches in the largest multicenter treatment-naïve UC cohort to date. We were thereby able to make several novel observations, while avoiding confounding effects of prior therapy and longstanding duration of disease^49,50^. We validated key gene signatures across several independent pediatric and adult cohorts. However, whole biopsies, composed of a mixture of cellular components, were used rather than single-cell transcriptomics. To address this limitation, we performed computational deconvolution of cell subset proportions in UC and controls, and across UC severity with specific differences noted. Deconvolution has limitations as it only estimates cell subset proportions and it is possible that closely related cell types will not be discriminated. Future studies using single-cell preparations, prioritized by the current dataset, will be important for further cellular subset characterizations. While we noted only monocytes to vary with corticosteroid response, its proportion score was not significant when added to the outcome model in place of the gene signature. By analyzing published datasets obtained from IEC isolated from treatment-naïve pediatric UC patients and by FACs analyses gating on EpCAM+ epithelial cells, we demonstrate that the mitochondrial gene expression signature and function respectively are suppressed in the epithelial compartment in UC.

In summary, our UC transcriptomics cohort is the largest and most comprehensive to date and the only data set to utilize pretreatment samples, and to link these to 16S microbial community data and response to standardized first-line corticosteroid therapy. We implicate a robust colonic mitochondriopathy in overall UC pathogenesis. Already at diagnosis genes associated with UC severity are enriched for those known to drive epithelial transformation. A validated corticosteroid response gene signature and higher anti-inflammatory *ALOX15* expression are associated with higher odds of achieving early clinical remission, with remarkable overlap with genes implicated in response to biologics. A shift to personalized approaches targeting specific mechanisms in individual patients will be key to reducing the increasing disease burden of UC worldwide.

## Methods

### Study design and participants

PROTECT was a multicenter inception cohort study^3^ based at 29 centers in the USA and Canada. Children aged 4–17 years with a diagnosis of UC based on accepted clinical, endoscopic, and histological parameters^5^, disease extent beyond the rectum, a baseline Pediatric Ulcerative Colitis Activity Index (PUCAI) score of at least 10, no previous therapy for colitis, and stool culture negative for enteric bacterial pathogens and *Clostridium difficile* toxin were included. Informed consent or assent was obtained in all cases and the study was approved by the local investigational review board at all investigative sites. Detailed protocol and study description can be found in Hyams et al.^3^. Disease extent was classified as proctosigmoiditis, left-sided colitis (to the splenic flexure), extensive colitis (to the hepatic flexure), or pancolitis (beyond the hepatic flexure) by visual evidence. Patients with severe or fulminant disease at presentation who received a flexible sigmoidoscopy because of safety concerns were assigned to the extensive colitis group (unassessable). Clinical activity at diagnosis was established with the PUCAI (range 0−85), Mayo endoscopic scope (grade 1–3), and total Mayo score (range 0−12). PUCAI less than 10 denoted inactive disease or remission, 10−30 denoted mild disease, 35−60 denoted moderate disease, and 65 or higher denoted severe disease. A central pathologist blinded to clinical data examined a single rectal biopsy from each patient and assessed histological features of chronicity and quantitated acute inflammation. Paneth cell metaplasia, surface villiform changes, or basal lymphoid aggregates were recorded if present. The description of eosinophilic inflammation included the peak number of eosinophils per high-power field relative to a cut-point (>32 cells per high-power field) derived from a study of normal rectal biopsies in children^3,5^.

Depending on initial PUCAI score, patients received initial treatment with either mesalamine (mild disease), or corticosteroids (moderate and severe disease), with some physician discretion allowed. A detailed description of treatment guidelines is provided in Hyams et al.^3^. All patients on mesalamine received study-supplied Pentasa (Shire Pharmaceuticals/Pantheon, Greenville, NC, USA). For this part of the study we used a week 4 (W4) remission outcome defined as PUCAI < 10 without additional therapy or colectomy. Twenty additional Cincinnati Children’s Hospital Medical Center patients were enrolled under another IRB approved protocol, and were included in the current analyses as non-IBD controls after clinical endoscopic, and biopsies evaluation demonstrated no histologic and endoscopic inflammation. Rectal mucosal biopsies from a representative subcohort of 206 PROTECT UC patients and 20 age and gender matched non-IBD controls underwent high coverage transcriptomic profiling using Illumina RNAseq (Table 1). These constituted the Discovery cohort for the current study.

The representative subcohort for RNAseq was defined by having a baseline rectal biopsy available to be included in the RNAseq analysis, and must also have the following data available in order to be assigned to the appropriate clinical subgroup: baseline PUCAI, medication data including the need for rescue or colectomy through week 4 and a week 4 PUCAI if the participant has not required rescue or a colectomy during the first 4 weeks. The following PROTECT participants were not eligible for the RNAseq analysis: patients with a diagnosis other than UC after enrollment, patients with significant baseline violations, patients who took rescue medications for a non-UC reason within the first 4 weeks, baseline RNA sample is unavailable, race is either “Asian”, “Black or African American” or “Unknown”, baseline PUCAI < 35 but did not start on mesalamine as first therapy, baseline PUCAI ≥ 35 but did not start on corticosteroids as first therapy. A total of 219 were selected, and data for 206 were ultimately available, after excluding 5 subjects based on the RNAseq data as described below, and 8 with insufficient RNA.

### Rectal RNA extraction and RNAseq analysis

RNA was isolated from rectal biopsies obtained during diagnostic colonoscopy using the Qiagen AllPrep RNA/DNA Mini Kit. PolyA-RNA selection, fragmentation, cDNA synthesis, adaptor ligation, TruSeq RNA sample library preparation (Illumina, San Diego, CA), and paired-end 75 bp sequencing was performed^9^. An additional validation of the baseline rectal gene expression at diagnosis utilized the independent RISK cohort of treatment-naïve pediatric patients (55 non-IBD controls, 43 UC patients, and 92 CD patients with rectal inflammation) and single-end 75 bp mRNA sequencing^9^. Reads were quantified by kallisto^51^, using Gencode v24 as the reference genome and transcripts per million (TPM) as an output. We included 14,085 protein-coding mRNA genes with TPM above 1 in 20% of the samples in our downstream analysis. Only samples for which the gene expression (Y encoded genes and XIST)-determined gender matched the clinical-reported gender were included in the analyses (we excluded only one sample with unmatched gender). Four other PROTECT samples were excluded due to poor read quality. A total of 226 RNAseq samples with mean read depth of ~47 M (14 M std. deviation) were stratified into specific clinical subgroups including Ctl (*n* = 20), and UC (*n* = 206), and were substratified based on disease severity, and on histologic findings. Differentially expressed genes were determined in GeneSpring® software with fold change differences (FC) ≥ 1.5 and using the Benjamini–Hochberg false discovery rate correction (FDR, 0.001) for all analyses except for the corticosteroid response genes that was calculated out of the 712 severity genes with FDR < 0.05. Unsupervised hierarchical clustering using Euclidean distance metric and Ward’s linkage rule was used to test for groups of rectal biopsies with similar patterns of gene expression. ToppGene^6^ and ToppCluster^7^ software were used to test for functional annotation enrichment analyses of immune cell types, pathways, phenotype, immune cell-type enrichments, and biologic functions. Visualization of the network was obtained using Cytoscape.v3.0.2^52^.

For validation of the association between baseline gene expression and outcome, we also generated independent Lexogen QuantSeq 3′ mRNAseq libraries^19^ and single-end 100 bp sequencing for 134 participants who also had Illumina mRNAseq data (the Discovery Cohort) and for 50 participants who did not have Illumina mRNAseq data (the independent Validation cohort, Table 1). PCA was performed to summarize variation in gene expression between patients, and principal components (PC) values were extracted for downstream analyses. We considered several central gene expression pathways PC1 preidentified by the previous differential expression analyses and functional annotation enrichment analyses of the core 5296 UC genes, the 712 severity genes, and the 115 corticosteroid response gene signature for the model building and associations with the microbial composition as described below. PROTECT (GSE109142) and RISK (GSE117993) rectal mRNAseq data sets were deposited into GEO.

### Analyses of microarrays

We obtained colon biopsy gene expression data and patient clinical data from published studies available in Gene Expression Omnibus (GEO) as summarized in Suppl. Table 1. The Affymetrix raw gene array data (.CEL files) were processed to obtain a log2 expression value for each gene probe set using the robust multichip average (RMA) method implemented in R; the Affymetrix GeneChip Human Genome U133 Plus 2.0 Arrays were processed in R with the affy package (v1.56.0) and the gcrma package (2.50.0), and the Human Gene 1.0 ST arrays were processed with the oligo package (v1.42.0). For comparative analysis, the LIMMA package^53^ was used to identify the filtered gene probe sets that showed significant differential expression between the studied groups, based on moderated t-statistics with Benjamini−Hochberg FDR correction for multiple testing. Gene probe sets were selected as biologically significant using FDR < 0.05 and a fold change (FC) ≥ 1.5. When genes in microarray data were represented by multiple probes, the probe with the greatest interquartile range was selected for analysis. PCA was performed on the normalized log2 microarray data of control and UC samples and PC1 values were calculated.

### Microbiome analyses

DNA was extracted from PROTECT UC stool samples and subjected to 16S rRNA amplicon sequencing. OTU clustering and taxonomic assignment was performed^24^ (NCBI SRA Bioproject: PRJNA436359). Briefly, for the OTU analysis the 16S bioBakery workflow built with AnADAMA2^54^ was applied and microbial taxonomy was based on the Greengenes 16S rDNA database (version 13.5)^55^. Samples were subsequently filtered (min 3000 reads and OTU prevalence threshold of 20 samples). Statistical significance was established using hierarchical all-against-all association testing (HAllA: http://huttenhower.sph.harvard.edu/halla) in all-against-all mode using Spearman as the similarity measure and a cutoff of 0.2 for the FDR. Overall, 156 PROTECT stools at baseline were available that also had mRNAseq data. In total, 149 OTUs were significantly associated with 9 genes, and 15 pathways, with 36 below FDR 0.1 (Supplementary Dataset 5). Overall, only 28 RISK CD cases and 21 PROTECT Lexogen UC validation cohort cases had both fecal microbial profiling and rectal mRNAseq data, providing insufficient power for validation of these results.

### Computational deconvolution

To estimate cell subset proportions, we performed a cell-type deconvolution. We utilized xCell^56^, a computational method that is able to infer 64 various cell types (e.g., immune cell types, epithelial, and stroma cell types) using gene signatures. To ensure robustness of our downstream analyses, we considered only cell types that had significant enrichment scores (FDR corrected *P* values < 0.1 in at least 80% of the samples). We calculated the significance using two approaches, and took into account cell types that were significant in at least one of them. The first includes randomization of the genes in the signatures used for generating the enrichment scores and the second includes using simulations where the tested cell type is not included in the mixture^56^. Epithelial cells were considered but did not vary significantly between samples. We identified the following significant cell types: active DC, astrocytes, B cells, CD4+ naive T cells, conventional DC, DC, memory B cells, plasma cells, Th1 cells, and monocytes. The scores of active DC and DC as well as B cells and “Memory B cells” across samples were positively and highly correlated (Supplementary Fig. 7) and we consider the more specific and biologically relevant activated DC and memory B cells. Astrocytes cell type was removed from the calculation.

### High-resolution respirometry

The Oxygraph-2k (O2k, Oroboros Instruments, Innsbruck, Austria) was used for measurements of respiration. Each chamber was air-calibrated in Mir05 respiration medium (0.5 mM Ethylenediaminetetraacetic acid (EDTA), 3 mM MgCl_2_, 60 mM k-lactobionic acid, 20 mM taurine, 10 mM KH_2_PO_4_, 20 mM 4-(2-hydroxyethyl)-1-piperazineethanesulfonic acid (HEPES), 110 mM d-sucrose, 0.1% Bovine serum albumin (BSA) essentially fatty acid free) before each experiment. All experiments were performed at 37 °C. Oxygen concentrations in each chamber never dropped below 80 μM during any experiment. Patient biopsies were taken from the cecum and rectum in both control patients (*N* = 5) and patients with UC (*N* = 9). Cecal and rectal biopsies were homogenized in Mir05 respiration medium, and 100 μl of the tissue homogenate was added to each chamber. Once baseline oxygen levels in each chamber became stable, cytochrome *c* (10 μM), malate (2 mM), pyruvate (5 mM), Adenosine diphosphate (ADP) (5 mM), and glutamate (10 mM) were added to stimulate respiration through Complex I. Once the oxygen consumption rate plateaued, succinate (10 mM) was added to assess the combined activity of Complexes I + II. Next, rotenone (1 mM) was added to inhibit Complex I activity, and additional succinate was added to analyze maximal Complex II activity. Carbonyl cyanide p-trifluoromethoxyphenylhydrazone (0.5 μM) was then added to uncouple the mitochondrial membrane and induce maximal respiration. Respiration rates were normalized to the amount of protein added for each sample. Complex I respiration was defined as the rate of respiration of malate/ADP/pyruvate/glutamate (first succinate—rotenone). Complex II respiration was defined as respiration after adding the second dose of succinate minus Complex I respiration. Average rates of oxygen consumption [(pmol/(s×ml)/μg protein] + standard error of the mean (SEM) were graphed.

### Cold enzyme biopsy prep to generate single cells

Colon biopsies were minced in a Petri dish on ice in the presence of Native Bacillus Licheniformis psychrophilic proteases at 1 mg/ml (Creative Enzymes, Shirley, NY), transferred to an Eppendorf tube, intermittently vortexed for 30–60 s, placed on ice, and gently pipetted over 15 min^57^. The suspension was centrifuged at 90 × *g* and the supernatant filtered over a 40 mcM filter. Additional enzyme was added to residual tissue and the procedure repeated for an additional 15 min. Cells were counted with trypan blue and 85−99% viability was noted.

### JC1 mitochondrial membrane potential measurement

JC1 staining was performed on the above single-cell isolations with flow cytometry using the JC-1 (5,5″,6,6″-tetrachloro-1,1″,3,3″-tetraethylbenzimidazolylcarbocyanine iodide, Molecular Probes, Inc. Eugene, OR) reagent according to the manufacturer’s instructions. In brief, JC-1 dye was added at 1 mcM to washed cells, and incubated for 20 min at 37 °C, 5% CO_2_. Cells were washed and CD45 APC-Cy7 (BD Bioscience, Franklin Lakes, NJ) and EpCAM APC (BioLegend, San Diego, CA) antibodies were added for an additional 30 min at room temperature. Cells were washed, acquired on a Canto flow cytometer, and data were analyzed using DeNovo software. The MMP was calculated as the ratio of PE-MFI/FITC-MFI in EpCAM+ and CD45+ cells. Representative data are shown in Supplemental Fig. 1. As a positive control for the specificity of the assay we used 50 mcM of CCCP (carbonyl cyanide 3-chlorophenylhydrazone) to depolarize the MMP measured using the JC-1 dye.

### Immunohistochemistry

Immunohistochemistry detection of MT-CO1, COX5A, and REG1A was performed^32,58^, using anti-Complex IV subunit I (Thermo Fisher Scientific cat. #459600), anti-Complex IV subunit Va (Thermo Fisher Scientific cat. #459120), and anti–REG1A (R&D Systems, INC. cat. #MAB4937). Staining was examined using an Olympus BX51 light microscope and digitally recorded at ×20 and ×40 magnification.

### Regression analysis for week 4 remission

We used multiple logistic regression to (1) determine the prognostic power of baseline clinical information, and (2) assess additional prognostic power resulting from including baseline gene expression in predicting remission 4 weeks after diagnosis in the moderate-severe group that received initial corticosteroid therapy. Pairwise association testing was performed to identify baseline variables appropriate for model building (nominal *P* value < 0.05). Clinical information considered for inclusion in the models were baseline clinical and endoscopic severity (Total Mayo EEF), Paris and Montreal classifications, presence of >32 eosinophils in the baseline rectal biopsy, gender, race, age at diagnosis, baseline BMI *z*-score, and serum albumin. We considered the corticosteroid response genes PC1 and several other central genes pathways PC1 preidentified by the previous differential expression analyses and functional annotation enrichment analyses of the core 5296 UC genes and the 712 severity genes. The corticosteroid response gene signature passed our predefined expression filtering with the highest significance. For validation of the within subject biopsy consistency, we performed in parallel mRNAseq of paired biopsies obtained at the same time as the rectal sample used to derive the predictive gene panel in a subset of patients (*n* = 6). Those comparisons showed a strong correlation of 0.94 (*P* = 0.005) for the corticosteroid response gene signature PC1 between pairs of biopsies. Using forward selection, we progressively constructed several logistic regression models that respectively include clinical and endoscopic severity, eosinophilic grade, and sex (model 1), and clinical and endoscopic severity, eosinophilic grade, sex, and the corticosteroid response gene signature PC1 (model 2). In model 3 we tested how well eosinophil-associated genes can replace the histologic eosinophil grade in model 2. At each step of model building, variables with *P* < 0.1 were considered for inclusion; a likelihood ratio test was performed to compare the model with and without the new variable. Each new variable with likelihood ratio *P* < 0.05 was maintained in the model. The reliability of the final model was tested by tenfold cross validation. Model fit and improvement at each stage was assessed using AUC, Akaike Information Criterion (which penalizes for model complexity), and sensitivity and specificity.

### Summary of statistical tests used

Shapiro−Wilk normality test was used on the continuous clinical parameters, and on specific gene expression, and PC1. If the data were not normally distributed, Mann−Whitney was used to compare two groups, and Kruskal−Wallis with Dunn’s Multiple Comparison test was used for comparison of more than two groups. However, if the data were normally distributed unpaired *t* test was used to compare two groups, and ANOVA with FDR was used for comparison of more than two groups. *All two-sided *P* < 0.05, **P < 0.01, ***P < 0.001. All statistical analyses were performed in SASv9.3 or GraphPad Prism v7.04.

### Study approval

This study was approved by the Institutional Review Boards at each of the participating PROTECT sites. Informed consent was obtained for all participants, with assent obtained for those aged 11 and older.

## Supplementary information

Supplementary information

Peer Review File

Description of Additional Supplementary Files

Supplementary Data 1

Supplementary Data 2

Supplementary Data 3

Supplementary Data 4

Supplementary Data 5

## Data Availability

PROTECT (GSE109142) and RISK (GSE117993) rectal mRNAseq data sets were deposited into GEO.
