# Supplementary material for: Ulcerative colitis mucosal transcriptomes reveal mitochondriopathy and personalized mechanisms underlying disease severity and treatment response
Source: Nat Commun. 2019 Jan 3;10:38. doi: 10.1038/s41467-018-07841-3 (PMC6318335; doi:10.1038/s41467-018-07841-3)
Supplement: Supplementary file 1 — Supplementary information [file 41467_2018_7841_MOESM1_ESM.pdf]

Supplementary Tables:

Supplementary Table 1 (page S-2): Summary of UC transcriptomic datasets and their use for validation of the current PROTECT study results.

Supplementary Table 2 (page S-3): Histologic severity score

Supplementary Table 3a (page S-4): Baseline clinical, demographic, and gene expression characteristics grouped by Week 4 outcome.

Supplementary Table 3b: Multivariable models associated with week 4 remission in 147 patients (as in Table 1) with moderate-severe disease that received corticosteroids using & adjusting for the histology severity score.

Supplementary Table 3c: Multivariable models associated with week 4 remission in 147 patients (as in Table 1) with moderate-severe disease that received corticosteroids using & adjusting for the computational deconvolution monocyte proportion score.

Supplementary Figures.

Supplemental Figure 1 (page S-6): The mitochondrial membrane potential is reduced in active UC epithelial cells.

Supplemental Figure 2 (page S-7): The core rectal gene signatures associated with clinical and mucosal disease severity.

Supplementary Figure 3 (page S-8): Rectal REG1A staining.

Supplementary Figure 4 (page S-9): Immune cell type enrichment analyses using ToppGene.

Supplementary Figure 5 (page S-10): Corticosteroid response gene signature PC1 is associated with week 4 outcome.

Supplementary Figure 6 (page S-11): Regression model bootstrapping.

Supplementary Figure 7 (page S-12): Correlation heat map of the most significant cell types of the cellular deconvolution analysis.

Supplementary Table 1 Summary of UC transcriptomic datasets and their use for validation of the current PROTECT study results. Data sets were chosen for validation based on data availability and cohort design and size.

Transcriptomic Study	Colon samples #	Publication	Platform	Method	Deposition	Used here for discovery/validation
PROTECT pediatric UC inception	206 UC<br>20 Ctl	Current paper	RNAseq	Illumina TruSeq mRNAseq	GSE109142	Current study discovery cohort
RISK pediatric IBD inception	43 UC<br>93 CD<br>55 Ctl	Current paper<br>Haberman et al, JCI 2014[1]	RNAseq	Illumina TruSeq mRNAseq	GSE117993	Yes, to validate UC signature. Test CD types signature
Adult IBD on therapy	74 UC active<br>23 UC inactive<br>8 active CD<br>11 Ctl	Vanhove, W. *et al.* IBD J 2015[2]	Microarray	Affymetrix GeneChip Human Gene 1.0 ST array	GSE59071*	Yes, to validate active UC and active CD signatures. Test inactive UC signature
Adult IBD on therapy	24 UC<br>19 cCD<br>19iCD<br>18 Ctl	*Arjit, PLoS One,* 2009[3]<br><br>Used by other studies including:<br>West Nat Med, 2017[4]<br>Gaujoux, Gut 2018[5]	Microarray	Affymetrix GeneChip Human Genome U133 Plus 2.0 Arrays	GSE16879	Yes, to test PROTECT corticosteroid refractory gene signature in anti-TNF refractory UC
Adult UC on therapy	24 UC cohort A<br>22 UC cohort B	Arjit Gut 2009[6]	Microarray	Affymetrix GeneChip Human Genome U133 Plus 2.0 Arrays	GSE14580 – cohort A<br>GSE12251 – cohort B	
Adult UC on therapy	15 UC active<br>8 UC remission<br>13 control	Planell Gut, 2013[7]	Microarray	Affymetrix GeneChip Human Genome U133 Plus 2.0 Arrays	GSE38713	
Adult UC on therapy	48 UC<br>0 Ctl	Toedter AJG 2011[8]	Microarray	Affymetrix GeneChip Human Genome U133 Plus 2.0 Arrays	GSE23597.	
Adult IBD on therapy	37 UC active<br>44 UC not active<br>20 Ctl	Granlund, Plos one 2013[9]	Microarray	llumina human HT-12 expression BeadChips	ArrayExpress E-MTAB-184	
Adult UC on therapy	67 UC time 0<br>40 UC with WK6/12 outcome<br>12 Ctl	*Arijs Gut* 2018[10]	Microarray	Affymetrix Human Gene 1.0 ST arrays	GSE73661*	Yes, to test PROTECT corticosteroid refractory gene signature in anti-integrin $\alpha_4\beta_7$ refractory UC
UC adults new onset	14 new onset UC<br>16 Ctl	Taman, JCC 2018[11]	mRNAseq	Illumina TruSeq mRNAseq	Not deposited	
IBD pediatric new onset	11 UC<br>11 CD<br>11 Ctl	Howell, Gastro 2018[12]	mRNAseq	Illumina TruSeq mRNAseq	E-MTAB-5464	Yes, to validate UC signature in isolated epithelia

*We also compared differentially expression (DE) genes between UC vs. Controls in GSE73661 and there was an overlap of 95% with DE genes in GSE59071

Supplementary Table 2 Histologic severity score.

Grade	Rectal Biopsy Histologic Feature
0	No inflammation
1	Chronic inflammation only
2	Mild acute neutrophil inflammation – no crypt abscesses
3	Moderate to marked acute neutrophil inflammation with crypt abscesses
4	Mucosal ulcers and erosions

Supplementary Table 3a Baseline clinical, demographic, and gene expression characteristics in moderate-to-severe patients grouped by week 4 outcome.

Week 4 evaluable population	Week 4 remission (n=75)	Week 4 no remission (n=77)	p-value
Age (years)<br>(Median, IQR)	14 (10-15)	14 (11-16)	0.517
Female (%)	42(56%)	30 (39%)	0.035
**Baseline characteristics:**			
Total Mayo Score<br>(range 0-12)<br>(Median, IQR)	8 (7-10)	10 (8-11)	0.0003
Albumin<br>(g/dL) (Median, IQR)	3.8 (3.3-4.1)	3.6 (2.9-4)	0.133
Rectal Eosinophil Level<br>(count > 32 /hpf)	49/73 (67%)	31/74 (42%)	0.003
Baseline fecal calprotectin<br>(Median, IQR)	n=58<br>2812(1498-4046)	n=50<br>2776(1396-3991)	0.716
Corticosteroid Response Gene Signature (PC1 z score values)<br>(Median, IQR)	0.169 (-0.163-0.662)	0.691 (0.159-1.149)	<0.0001
ALOX15 Gene Exp. (TPM)<br>(Median, IQR)	0.8 (0.5-1.5)	0.5 (0.3-0.8)	0.0002

Baseline variables were compared between remission/non-remission in moderate-severe patients using non-parametric Mann-Whitney tests and chi-square tests where appropriate. IQR: interquartile range

Supplementary Table 3b Multivariable models associated with week 4 remission in 147 patients with moderate-severe disease that received corticosteroids using & adjusting for the histology severity score.

Model Variables	OR (95% CI)	Variable P	Model AIC	Model AUC	Model ChiSq	Model P
Total Mayo Score (range 0-12)	0.72 (0.572, 0.989)	0.004	179.97	**75.9 (67.9-839)**	33.80	<0.0001
ALOX15 Gene Exp. (TPM)	2.97 (1.398, 6.293)	0.005				
Sex (M vs F)	0.45 (0.213, 0.932)	0.031				
Histologic severity score	0.855 (0.53, 1.379)	0.520				
Total Mayo Score (range 0-12)	0.78 (0.618, 0.99)	0.041	173.94	**77.4 (69.7-85.1)**	49.74	<0.0001
ALOX15 Gene Exp. (TPM)	2.78 (1.322. 5.837)	0.007				
Sex (M vs F)	0.43 (0.203, 0.92)	0.030				
Corticosteroid Response Gene Signature (PC1 z-score values) after adjusting for histologic severity score	0.42 (0.207, 0.835)	0.014				

OR: odds ratio; AIC: Akaike's information criterion; AUC: area under the ROC curve; LR: likelihood ratio; ROC: Receiver Operator Characteristic.

Supplementary Table 3c Multivariable models associated with Week 4 remission in 147 patients with moderate-severe disease that received corticosteroids using & adjusting for the computational deconvolution monocyte proportion score.

Model Variables	OR (95% CI)	Variable P	Model AIC	Model AUC	Model ChiSq	Model P
Total Mayo Score (range 0-12)	0.71 (0.563, 0.899)	0.0044	180.39	**75.7 (67.7-83.7)**	33.39	<0.0001
ALOX15 Gene Exp. (TPM)	3.07 (1.458, 6.467)	0.0032				
Sex (M vs F)	0.44 (0.209, 0.906)	0.0263				
Monocyte proportion score	1.01 (0.001, 673.12)	0.997				
Total Mayo Score (range 0-12)	0.73 (0.58, 0.932)	0.0111	168.49	**79.8 (72.5-87.1)**	45.28	<0.0001
ALOX15 Gene Exp. (TPM)	2.91 (1.346, 6.306)	0.0066				
Sex (M vs F)	0.47 (0.219, 1.017)	0.055				
Corticosteroid Response Gene Signature (PC1 z-score values) after adjusting for monocyte proportion score	0.22 (0.087, 0.557)	0.0014				

OR: odds ratio; AIC: Akaike's information criterion; AUC: area under the ROC curve; LR: likelihood ratio; ROC: Receiver Operator Characteristic.

Supplementary Figures.

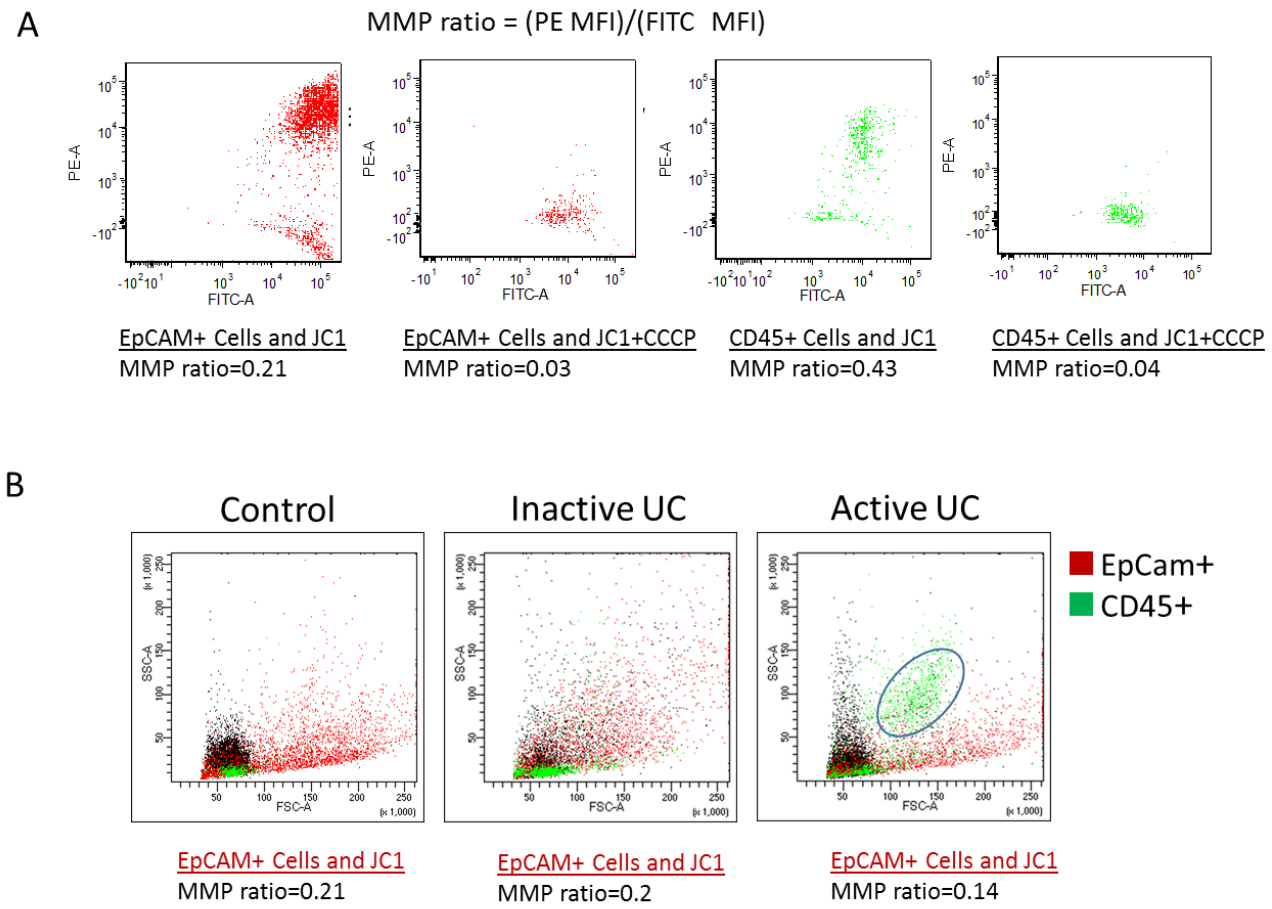

Supplementary Figure 1: The mitochondrial membrane potential is reduced in active UC epithelial cells. The mitochondrial membrane potential (MMP) in EpCAM+ epithelial cells and CD45+ leukocytes isolated from colon biopsies was measured using JC1 staining of rectal biopsy single cell preps and flow cytometry as shown (5,5",6,6"-tetrachloro-1,1",3,3"-tetraethylbenzimidazolylcarbocyanine iodide, Molecular Probes, Inc.). (A) As a positive control we stained cells with 1mcM JC1 with and without the addition of 50mcM of the depolarizing agent CCCP (carbonyl cyanide 3-chlorophenylhydrazone). In the JC1+CCCP cells there is a substantial reduction in the MMP, confirming the specificity of the JC1 alone result. The MMP was calculated as the ratio of PE-MFI/FITC-MFI in EpCAM+ and CD45+ cells. (B) Representative FACS analyses of rectal biopsy single cell preps are shown illustrating the EpCAM+ epithelial and CD45+ leukocyte populations, with a marked increase in CD45+ cells in the active UC inflamed tissue. Mean fractions of control EpCAM+ epithelial cells and CD45+ leukocytes were 82% and 18%, in inactive UC were 71% and 29%, and in active UC 39% and 61%, respectively.

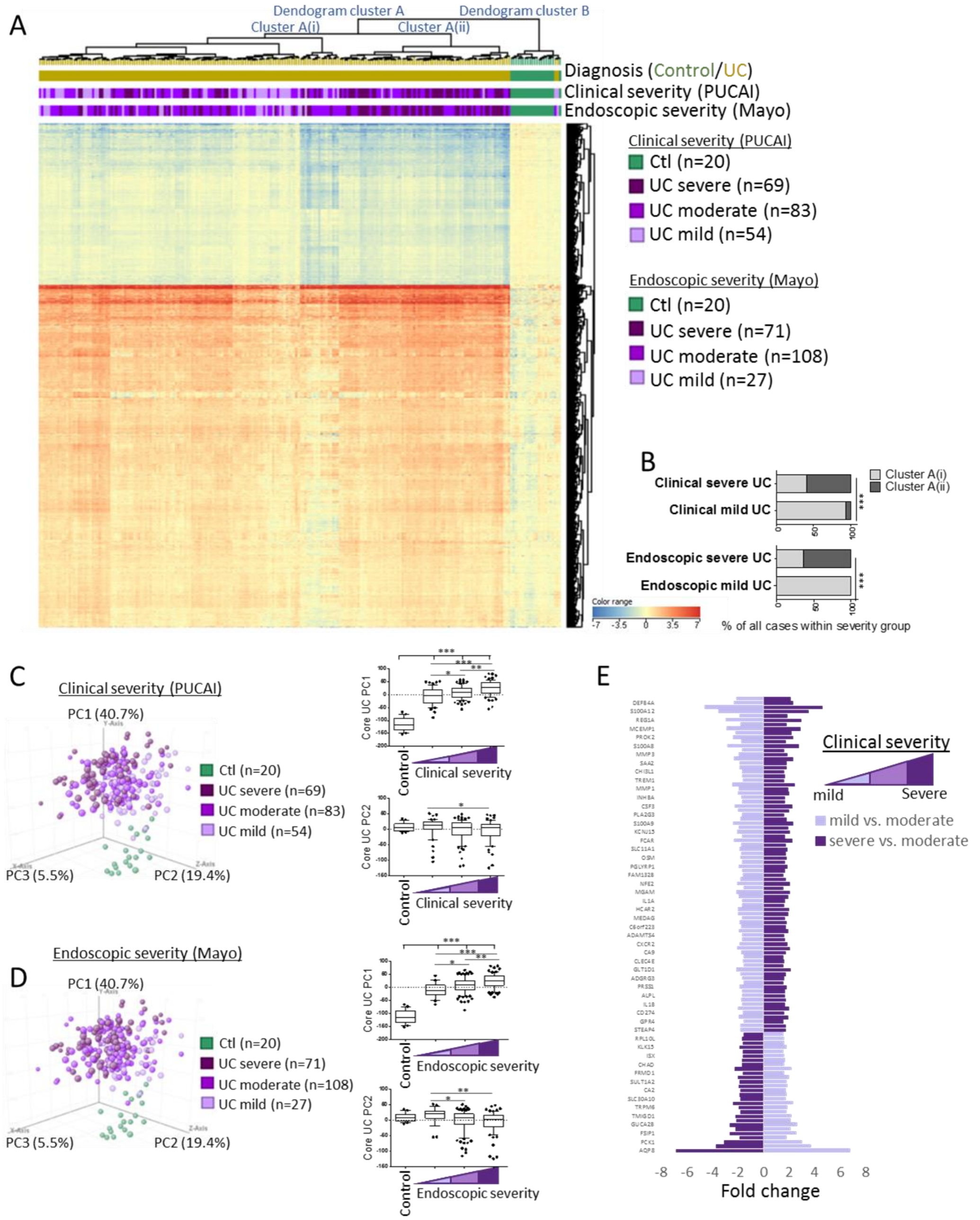

Supplementary Figure 2: The core rectal gene signatures associated with clinical and mucosal disease severity. (A) Hierarchical clustering of the 5296 differentially expressed rectal genes between 206 UC and 20 Ctl is visualized as a heat map with genes up-regulated compared to controls in red and genes down-regulated compared to controls in blue. Above the heat map, individual Ctl (green) and UC (mustard) samples are indicated and the different clinical and endoscopic severity cases in purple, where mild cases are indicated in light purple, severe cases in dark purple, and moderate in between. Dendrogram main branches are marked in A (left) and B (right) and the sub branching as A(i) and A(ii). (B) Frequency of mild or severe clinical or endoscopic cases in A(i) and A(ii) is shown. ***$P < 0.001$ using Chi square test. (C, D) 5296 differentially expressed core UC genes were used to view Ctl (green) and UC (purple) patients' samples separation on a principal component analysis (PCoA plot). Mild UC cases (clinical or endoscopic) are indicated in light purple, severe cases in dark purple, and moderate in between. The box and whiskers (10-90 percentiles) plots show samples loading PC1 or PC2 values stratified by Ctl, and by UC clinical or endoscopic severity using the 5296 genes. (E) Fold change of differentially expressed gene between severe (n=69) and moderate (n=83) clinical cases, and between mild (n=54) and moderate (n=83) clinical UC cases are shown with a stepwise alteration in gene expression in association with clinical severity. *$P < 0.05$, **$P < 0.01$, ***$P < 0.001$ using ANOVA with false discovery rate (FDR) for PC1 and Kruskal-Wallis with Dunn's Multiple Comparison test for PC2.

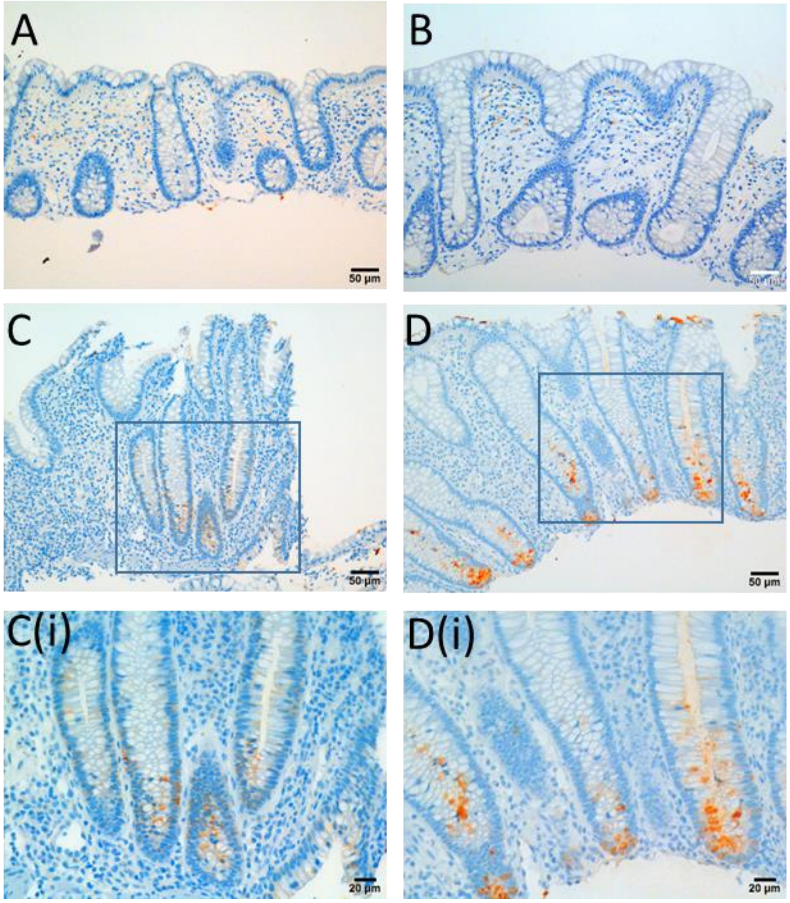

Supplementary Figure 3: Rectal REG1A Staining. REG1A rectal biopsy immunohistochemistry is presented for representative UC patients and non-IBD controls. No REG1A staining was detected in non-IBD controls with anti-REG1A antibody staining (A, B). Patients with moderate (C) and severe (D) UC at baseline respectively showing REG1A staining that correlated with transcripts per million (TPM) values of 827 and 1617 for gene expression by RNASeq, respectively. Images were captured using an Olympus BX51 light microscope and digitally recorded at 20x magnification. C(i) and D(i) are inset of C and D respectively that were recorded at 40x magnification.

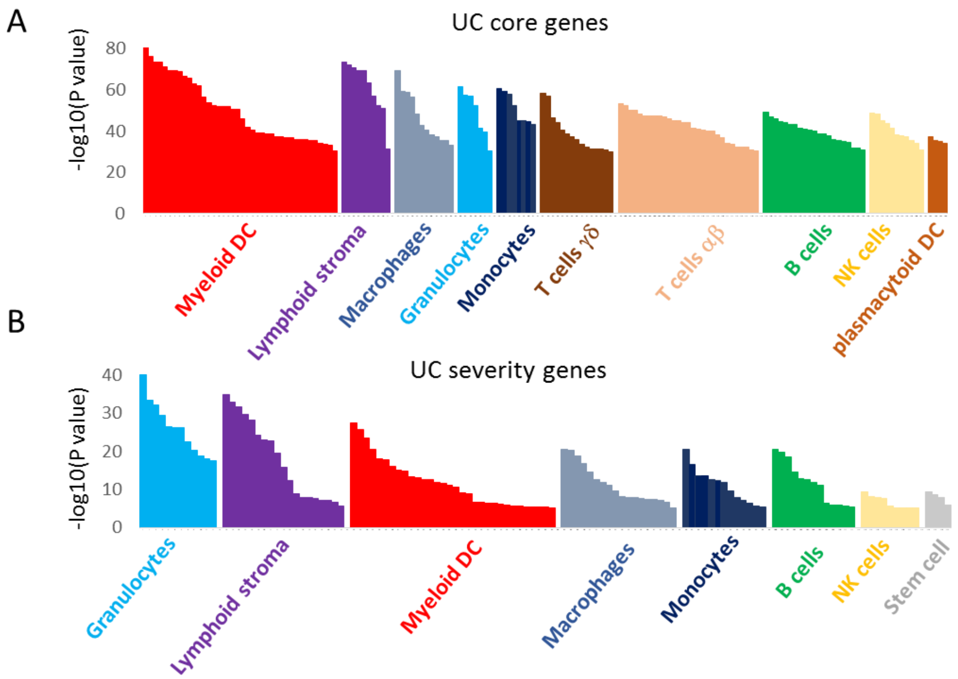

Supplementary Figure 4: Immune cell type enrichment analyses using ToppGene. Immune cell type enrichment of up-regulated genes for (A) 5296 core UC and (B) 712 UC severity genes using the Immunological Genome Project data series as a reference through ToppGene [6]. Enrichment for a given immune cell class is illustrated by colored bars on the x axis, with the significance for each individual cell subtype within the class shown as the –log10(P value) on the y axis. DC; Dendritic cells.

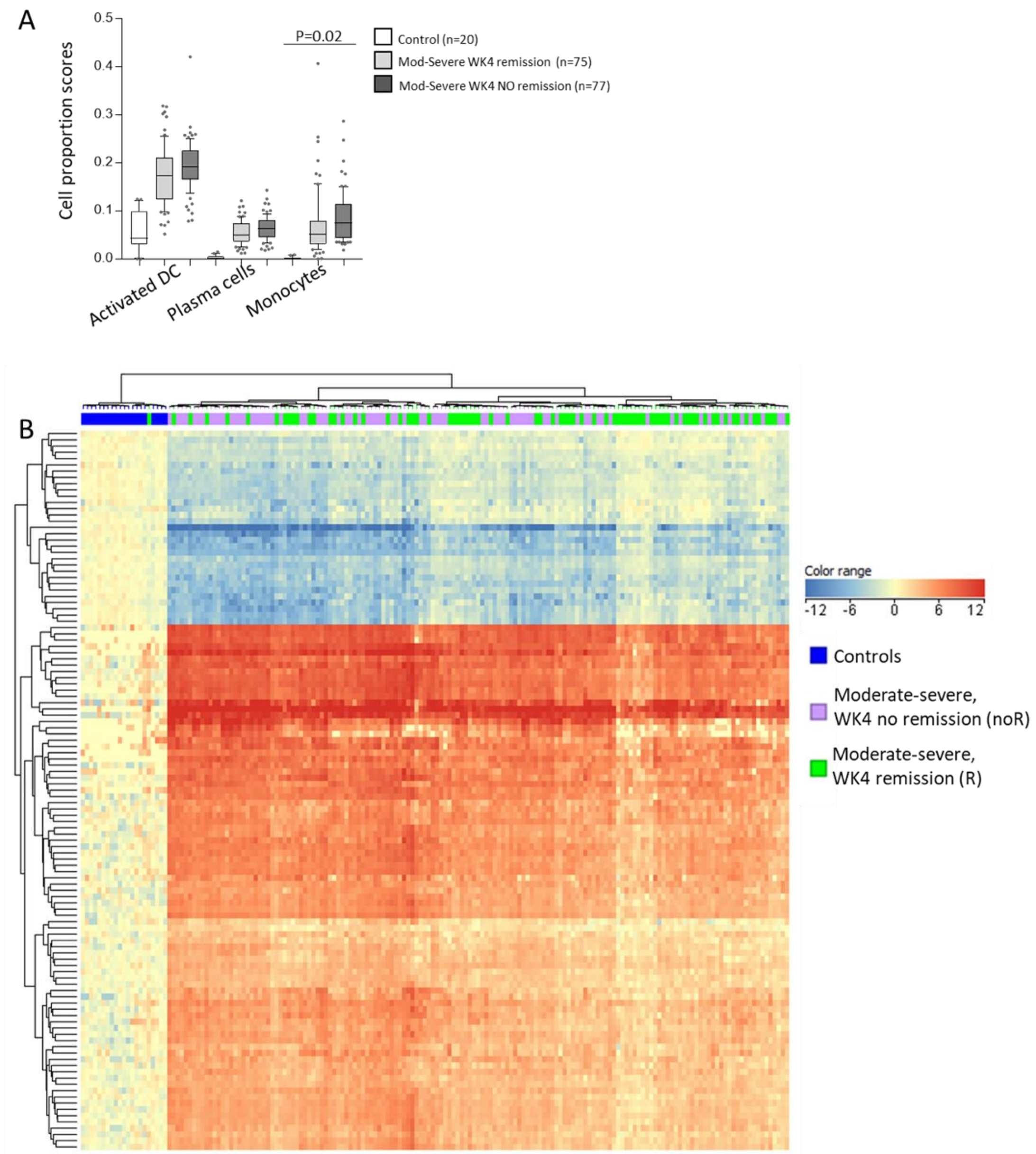

Supplementary Figure 5: The corticosteroid response gene signature. The corticosteroid response gene signature (115 genes) originated from differential expression between moderate-severe patients that achieved Week 4 (Wk4) remission and those that did not of the 712 severity genes. The complete gene list with functional annotation enrichment analyses of these 115 genes can be found in Supplementary Table 6. (A) Computational deconvolution analysis of cell subset proportions in controls and moderate-severe UC patients that did or did not achieve week 4 remission within the cells which increase with severity as shown in Figure 3. Only the monocyte cell proportion exhibited a significant difference between UC patients stratified by week 4 remission in Kruskal-Wallis with Dunn's Multiple Comparison test. (B) A heat map of the hierarchical clustered gene expression for the corticosteroid response gene signature for the indicated clinical subgroups is shown. Box and whisker plot with central line indicating median, box ends representing upper and lower quartile, and whisker represent 10-90 percentile.

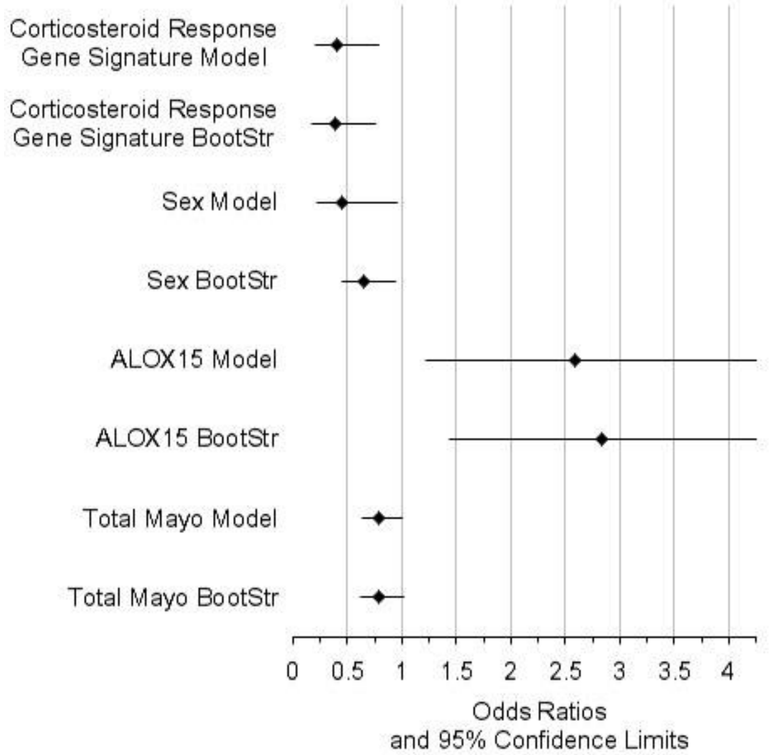

Supplementary Figure 6: Regression model bootstrapping. Results from bootstrapping and multiple imputation are shown for the final selected model for week 4 remission in moderate-to-severe UC patients based on overlap of model point estimates and confidence intervals with bootstrapped 95% confidence limits.

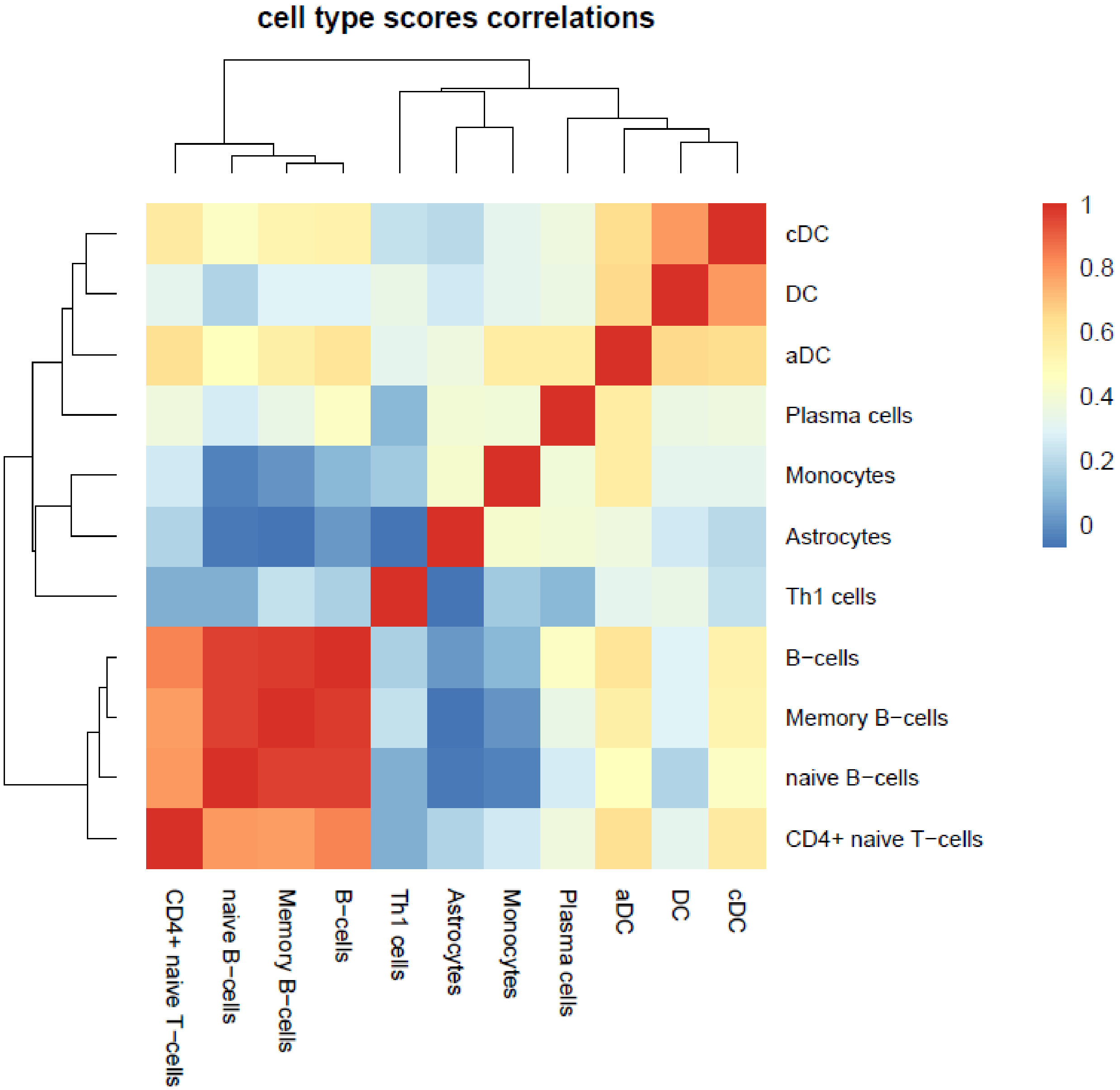

Supplementary Figure 7: Correlation heat map of the most significant cell types of the cellular deconvolution analysis. The following significant cell types: active Dendritic Cells, Astrocytes, B-cells, CD4+ naive T-cells, Conventional dendritic cells, Dendritic Cells, Memory B-cells, Plasma cells, Th1 cells, and Monocytes resulted from the cell-type deconvolution analyses. The scores of active Dendritic Cells and Dendritic cells as well as B-cells and "Memory B-cells" across samples were positively and highly correlated and we consider the more specific and biologically relevant activated DC and Memory B-cells for downstream analyses. Astrocytes cell type was removed from the calculation.

References for the online supplementary material

1 Haberman, Y. *et al.* Pediatric Crohn disease patients exhibit specific ileal transcriptome and microbiome signature. *The Journal of clinical investigation* **124**, 3617-3633, doi:10.1172/JCI75436 (2014).

2 Vanhove, W. *et al.* Strong Upregulation of AIM2 and IFI16 Inflammasomes in the Mucosa of Patients with Active Inflammatory Bowel Disease. *Inflammatory bowel diseases* **21**, 2673-2682, doi:10.1097/MIB.0000000000000535 (2015).

3 Arijs, I. *et al.* Mucosal gene expression of antimicrobial peptides in inflammatory bowel disease before and after first infliximab treatment. *PloS one* **4**, e7984, doi:10.1371/journal.pone.0007984 (2009).

4 West, N. R. *et al.* Oncostatin M drives intestinal inflammation and predicts response to tumor necrosis factor-neutralizing therapy in patients with inflammatory bowel disease. *Nat Med* **23**, 579-589, doi:10.1038/nm.4307 (2017).

5 Gaujoux, R. *et al.* Cell-centred meta-analysis reveals baseline predictors of anti-TNFalpha non-response in biopsy and blood of patients with IBD. *Gut*, doi:10.1136/gutjnl-2017-315494 (2018).

6 Arijs, I. *et al.* Mucosal gene signatures to predict response to infliximab in patients with ulcerative colitis. *Gut* **58**, 1612-1619, doi:10.1136/gut.2009.178665 (2009).

7 Planell, N. *et al.* Transcriptional analysis of the intestinal mucosa of patients with ulcerative colitis in remission reveals lasting epithelial cell alterations. *Gut* **62**, 967-976, doi:10.1136/gutjnl-2012-303333 (2013).

8 Toedter, G. *et al.* Gene expression profiling and response signatures associated with differential responses to infliximab treatment in ulcerative colitis. *The American journal of gastroenterology* **106**, 1272-1280, doi:10.1038/ajg.2011.83 (2011).

9 Granlund, A. *et al.* Whole genome gene expression meta-analysis of inflammatory bowel disease colon mucosa demonstrates lack of major differences between Crohn's disease and ulcerative colitis. *PloS one* **8**, e56818, doi:10.1371/journal.pone.0056818 (2013).

10 Arijs, I. *et al.* Effect of vedolizumab (anti-alpha4beta7-integrin) therapy on histological healing and mucosal gene expression in patients with UC. *Gut* **67**, 43-52, doi:10.1136/gutjnl-2016-312293 (2018).

11 Taman, H. *et al.* Transcriptomic Landscape of Treatment-Naive Ulcerative Colitis. *J Crohns Colitis* **12**, 327-336, doi:10.1093/ecco-jcc/jjx139 (2018).

12 Howell, K. J. *et al.* DNA Methylation and Transcription Patterns in Intestinal Epithelial Cells From Pediatric Patients With Inflammatory Bowel Diseases Differentiate Disease Subtypes and Associate With Outcome. *Gastroenterology* **154**, 585-598, doi:10.1053/j.gastro.2017.10.007 (2018).
