# Supplementary material for: Ulcerative colitis mucosal transcriptomes reveal mitochondriopathy and personalized mechanisms underlying disease severity and treatment response
Source: Nat Commun. 2019 Jan 3;10:38. doi: 10.1038/s41467-018-07841-3 (PMC6318335; doi:10.1038/s41467-018-07841-3)
Supplement: Supplementary file 3 — Description of Additional Supplementary Files [file 41467_2018_7841_MOESM3_ESM.pdf]

Description of Additional Supplementary Files

File Name: Supplementary Data 1
Description: The PROTECT core 5296 genes and functional annotation enrichments analyses, together with gene set replication in the RISK, adult UC GSE590711 , and IEC UC cohorts2 .

File Name: Supplementary Data 2
Description: The 712 UC severity genes and functional annotation enrichments analyses including immune cell types.

File Name: Supplementary Data 3
Description: The 187 surface villiform changes associated genes and functional annotation enrichments analyses.

File Name: Supplementary Data 4
Description: The 115 corticosteroid response genes and 50 anti-TNF response genes with functional annotation enrichments analyses.

File Name: Supplementary Data 5
Description: Results of the statistical significance correlations between genes and gene signatures and taxa as established by hierarchical all-against-all association testing [HAllA: (http://huttenhower.sph.harvard.edu/halla)]
