# Supplementary material for: Ulcerative colitis mucosal transcriptomes reveal mitochondriopathy and personalized mechanisms underlying disease severity and treatment response
Source: Nat Commun. 2019 Jan 3;10:38. doi: 10.1038/s41467-018-07841-3 (PMC6318335; doi:10.1038/s41467-018-07841-3)
Supplement: Supplementary file 2 — Peer Review File [file 41467_2018_7841_MOESM2_ESM.pdf]

Editorial Note: This manuscript has been previously reviewed at another journal that is not operating a transparent peer review scheme. The manuscript was considered suitable for publication without further review at Nature Communications.
